# Combined High—Throughput Proteomics and Random Forest Machine-Learning Approach Differentiates and Classifies Metabolic, Immune, Signaling and ECM Intra-Tumor Heterogeneity of Colorectal Cancer

**DOI:** 10.3390/cells13161311

**Published:** 2024-08-06

**Authors:** Cristina Contini, Barbara Manconi, Alessandra Olianas, Giulia Guadalupi, Alessandra Schirru, Luigi Zorcolo, Massimo Castagnola, Irene Messana, Gavino Faa, Giacomo Diaz, Tiziana Cabras

**Affiliations:** 1 Department of Medical Sciences and Public Health, Statal University of Cagliari, 09042 Monserrato (CA), Italy; cristina.contini93@unica.it (C.C.); gavinofaa@gmail.com (G.F.); 2 Department of Life and Environmental Sciences, Statal University of Cagliari, 09042 Monserrato (CA), Italy; olianas@unica.it (A.O.); aschirru75@unica.it (A.S.); 3 Department of Surgical Sciences, Statal University of Cagliari, 09042 Monserrato (CA), Italy; giulia.guadalupi@unica.it (G.G.); lzorcolo@aoucagliari.it (L.Z.); 4 Laboratorio di Proteomica, Centro Europeo di Ricerca sul Cervello, IRCCS Fondazione Santa Lucia, 00143 Roma, Italy; maxcastagnola@outlook.it; 5 Istituto di Scienze e Tecnologie Chimiche “Giulio Natta”, Consiglio Nazionale delle Ricerche, 00168 Roma, Italy; imessana53@gmail.com; 6 Department of Biology, College of Science and Technology, Temple University, Philadelphia, PA 19122, USA; 7 Department of Biomedical Sciences, Statal University of Cagliari, 09042 Monserrato (CA), Italy; gdiaz@unica.it

**Keywords:** CRC proteomics, intra-tumor heterogeneity, extracellular matrix, ROS, S100A9, galectin-3, sorting nexin-18, GRASP-1, basigin, mitochondrial metabolism

## Abstract

Colorectal cancer (CRC) is a frequent, worldwide tumor described for its huge complexity, including inter-/intra-heterogeneity and tumor microenvironment (TME) variability. Intra-tumor heterogeneity and its connections with metabolic reprogramming and epithelial–mesenchymal transition (EMT) were investigated with explorative shotgun proteomics complemented by a Random Forest (RF) machine-learning approach. Deep and superficial tumor regions and distant-site non-tumor samples from the same patients (n = 16) were analyzed. Among the 2009 proteins analyzed, 91 proteins, including 23 novel potential CRC hallmarks, showed significant quantitative changes. In addition, a 98.4% accurate classification of the three analyzed tissues was obtained by RF using a set of 21 proteins. Subunit E1 of 2-oxoglutarate dehydrogenase (OGDH-E1) was the best classifying factor for the superficial tumor region, while sorting nexin-18 and coatomer-beta protein (beta-COP), implicated in protein trafficking, classified the deep region. Down- and up-regulations of metabolic checkpoints involved different proteins in superficial and deep tumors. Analogously to immune checkpoints affecting the TME, cytoskeleton and extracellular matrix (ECM) dynamics were crucial for EMT. Galectin-3, basigin, S100A9, and fibronectin involved in TME–CRC–ECM crosstalk were found to be differently variated in both tumor regions. Different metabolic strategies appeared to be adopted by the two CRC regions to uncouple the Krebs cycle and cytosolic glucose metabolism, promote lipogenesis, promote amino acid synthesis, down-regulate bioenergetics in mitochondria, and up-regulate oxidative stress. Finally, correlations with the Dukes stage and budding supported the finding of novel potential CRC hallmarks and therapeutic targets.

## 1. Introduction

Colorectal cancer (CRC) is the third most common cancer worldwide, accounting for approximately 10% of all cancer cases, and it is the second leading cause of cancer-related deaths worldwide [1]. The high capacity of CRC to infiltrate and develop metastasis has the consequence that 40% of patients with CRC already have metastasis in the liver at the time of diagnosis. The prognosis of CRC is closely related to the stage, but the classification of patients is affected by a great variability in response to therapy and clinical outcome. The CRC complexity increases in relation to the localization of the tumor, which determines a huge inter-tumor heterogeneity, and to the intra-tumor heterogeneity connected to the tumor microenvironment (TME) [2,3]. The heterogeneity typical of CRC is also associated with several oncogenic signaling pathways, among them the glucose-related pathways. Indeed, the glucose metabolic reprogramming of cancer cells appears to be implicated in the malignant progression of CRC [4,5]. This metabolic alteration seems to be associated with the epithelial–mesenchymal transition (EMT), which is considered the main event promoting the invasion and migration of CRC cells [4,5]. However, developing a full understanding of the molecular mechanisms associated with EMT in CRC is still a challenge, and despite decades of research, the process of tumor dissemination is insufficiently understood. In our previous studies [6,7], we suggested a key role for thymosin β4 and thymosin β10 in colorectal cancer progression and the promotion of cancer invasion. Accordingly with other studies, both thymosins might be considered good candidates for diagnostic/prognostic biomarkers and therapeutic targets [8]. Indeed, thymosin β4-triggered EMT is considered the main event inducing the invasion and migration of CRC cells [7,8]. The connection between intra-tumor heterogeneity and molecular mechanisms leading to EMT is one of the focal factors for the development of precision medicine in CRC [2], and it was highlighted by several previous studies at the transcriptomic and proteomic levels [3]. From this point of view, the discovery of clinically relevant protein biomarkers correlated with intra-tumor heterogeneity was suggested to be promising for the diagnosis, prognosis, and treatment of CRC [2]. Proteomic investigations were shown to be optimal strategies for highlighting differential protein profiles due to quantitative or qualitative (post-translational modifications (PTMs) and isoforms) variations, which can be correlated with a different regulation of biological processes during carcinogenesis and in various developing stages of CRC. Several proteomic strategies have been applied in the last ten years on colon tissues or body fluids to discover candidate biomarkers for risk prediction, diagnosis, prognosis, staging, and monitoring the treatment response in CRC [2,3,9,10,11]. This investigation was an exploratory proteomic study on CRC to discover novel potential protein hallmarks able to distinguish, with high specificity and sensitivity, the tumor tissue from the healthy colon mucosa and to classify the superficial and the internal deep regions of the tumor. The two tumor regions represent functionally and histologically different areas, as demonstrated in previous studies [2,6,7]. The superficial region is the area from which the tumor grows inward and expands. It is mainly related to cancer cell proliferation, which interfaces with the intestinal lumen and communicates with the gut microbiota. The deep region is the part of the tumor that infiltrates the healthy tissue layers of the intestinal wall. From this front of invasion, the budding of cancerous cells develops and the metastases mature and detach. The knowledge of the intra-heterogeneity between the surface and the deep region of CRC at the proteomic level could be of interest in finding possible therapeutic targets and hallmarks with diagnostic potential, which are useful in developing effective and specific therapies. To this aim, we applied a shotgun proteomic approach based on micro-HPLC separation coupled with high-resolution (HR) mass spectrometry analysis and label-free quantitation (LFQ). Mass spectrometry data were analyzed using nonparametric Mann–Whitney (MW) and Kruskal–Wallis (KW) tests to identify proteins with different abundance in the healthy colon mucosa and in the superficial and deep internal regions of the tumor. In addition, Random Forest (RF) was used to identify the proteins that most effectively discriminate between the three tissue regions at the level of single samples. Such an approach was in line with the purpose of exploring possible correlations between proteomic data and clinical outcomes to identify potential biomarkers for CRC diagnosis and prognosis and/or possible therapy targets. Moreover, an enrichment analysis of the proteomic data was applied to highlight the intra-tumor heterogeneity regarding the biological processes that underlie EMT, metabolism, extracellular matrix (ECM) changes, and immune regulation in the TME. Finally, the possible protein–protein interactions (PPIs) and topological features of all identified proteins were evaluated and the possible key role of the protein hallmarks identified by our proteomic investigation. All the findings obtained from this explorative proteomic study represent a stimulus for further studies aimed at validating the proposed hallmarks.

## 2. Materials and Methods

**Reagents and Instruments**

All chemicals, reagents and disposable items were of analytical grade and were purchased from Sigma–Aldrich/Merck (Darmstadt, Germany), Roche Diagnostics (Basel, Swisse), Pierce™ (Thermo Fisher Scientific, Waltham, MA, USA), and Agilent (Santa Clara, CA, USA). HPLC–HR–MS experiments were carried out using an Ultimate 3000 micro-HPLC (Dionex, Sunnyvale, CA, USA) equipped with a FLM-3000-Flow manager module and coupled with a nano-electrospray source to an LTQ-Orbitrap Elite MS apparatus (ThermoFisher Scientific, San Jose, CA, USA).

**Study subjects**

The study included sixteen patients undergoing surgical resection of colorectal tumors performed by the unit of Colorectal Surgery, Department of Surgical Sciences (Cagliari University). Ethics committee approval was obtained for the study (protocol number 2020/10912; code: EMIBIOCCOR), and full written consent forms were obtained from all participants. Patients with colon cancer were included if they exhibited budding margins with evident morphological signs of EMT. Clinical and demographic data of the sixteen patients are reported in Table 1: six females and ten males were included in the study (average age ± SD 69 ± 13, and 72 ± 9, respectively). In Table 1, the original identification number assigned to the patients at the moment of the surgery was used. Patients were classified as A, B, or C according with the Dukes stage: one patient was in stage A (carcinoma limited to colon mucosa), eleven patients were designated as stage B (carcinoma confined to the muscular layer), and four patients as stage C (metastases present in the regional lymph nodes) [12]. Regarding the tumor budding index, namely the presence of de-differentiated single cells or small clusters of up to five cells at the invasive front of CRC [13], these were absent (0) in two patients, slight (1) in six patients, discrete (2) in three patients, and marked (3) in five patients.

**Sample Preparation**

Three kinds of tissues were collected from each patient: (i) the surface layer of the tumor, named “S”; (ii) the deep region of the tumor, named “D”; (iii) the healthy colon mucosa, named “H”. The three different tissue samples were of different sizes (but not exceeding 1 × 1 cm). S and D region samples were obtained with a section parallel to the luminal surface, which divided the tumor sample into two approximately equal parts. H region samples were obtained from colon mucosa with normal histology at sites distant from the tumor. Corresponding parts of the tumor and of healthy colon mucosa were histologically analyzed, as is clinically routine.

Immediately after the surgical resection, fresh tissue samples were washed from blood residues with a physiological solution and dipped in 600 μL of protein extraction buffer (25 mM Hepes pH 7.8, 50 mM KCl, beta-Octyl-glucopyranoside 0.2%, 1 mM dithiothreitol) to be homogenized in an ice bath. To inhibit proteases, one Mini-Complete ™ tablet (Roche Diagnostics, Basel, Swisse) was added to 10 mL of buffer. Homogenization, performed with an Ultra Turrax apparatus, was followed by three sonication cycles of 5 min. Homogenized tissues were centrifuged at 19,000× *g*, 4 °C for 10 min. The supernatant fraction of each sample was collected, while the insoluble fractions obtained were suspended in 200 µL of hydro-organic solution (0.05% trifluoroacetic acid and 20% acetonitrile), sonicated for 2 cycles of 5 min, and finally centrifuged at 19,000× *g*, 4 °C, for 10 min. The second supernatant fractions were added to the first ones to obtain a unique extract of soluble proteins and peptides from each sample. Protein extracts were dialyzed under stirring in 40 mM ammonium bicarbonate pH 7.8, at 4 °C for 3 h, using a Float-A-Lyzer G2 dialysis device with a 500 Da molecular cutoff (Sigma–Aldrich/Merck, Darmstadt, Germany). 

After dialysis, samples were lyophilized, and the powder suspended in 200 µL of 40 mM ammonium bicarbonate pH 7.8. Two aliquots of 5µL of each sample were used to determine in duplicate the total protein concentration by a bicinchoninic acid assay (QuantiPro BCA micro-assay kit, from Sigma–Aldrich/Merck). Proper volumes of each sample were calculated to collect aliquots of 4 µg of total protein to submit to tryptic digestion. Cysteine reduction in proteins was achieved by adding 10 mM dithiothreitol in 100 mM ammonium bicarbonate pH 7.8 to each sample and incubation at 100 °C for 5 min, followed by 30 min at 65 °C. Alkylation of cysteines was achieved by incubation with 55 mM iodoacetamide in the same buffer in the dark for 45 min at room temperature. Highly specific trypsin MS Grade (Pierce™, Thermo Fisher Scientific, Waltham, MA, USA) at 0.5 μg/μL was prepared in 40 mM ammonium bicarbonate pH 7.8, and mixed with pure acetonitrile at ratio 10:1 *v*/*v*. Trypsin solution was added to each sample at a ratio of 1:50 *w*/*w* enzyme/substrate, and incubation occurred overnight at 37 °C. All the samples were then dried desalted with OMIX C18 pipette tips (Agilent, Santa Clara, CA, USA) following the manufacturer’s instructions. The concentration of total proteins was not determined after the desalting step. However, after this step all the samples were diluted in 40 µL of 0.1% formic acid considering that 4 µg of total proteins in each sample was used for the digestion. Thus, we assumed that the 10 µL of final solution analyzed by HPLC–HR–MS contained tryptic peptides generated from 1 µg of total protein.

**HPLC–HR–MS/MS analysis**

HPLC separation of tryptic peptides was performed immediately after the desalting step using a reversed phase Easy Spray C18 nano-column (ThermoFisher Scientific, San Jose, CA, USA) with a 150 mm length, 50 µm inner diameter, and 2 µm particle size. The mobile phases were as follows: A, aqueous 0.1% formic acid; B, 20% aqueous 0.1% formic acid/80% acetonitrile (*v*/*v*). Chromatographic separation occurred in 100 min with a flow rate of 0.3 µL/min and the following gradient: 0–3 min at 4%B, 50% B in 67 min, 80% B in 20 min, 90% B in 2 min, continuing 90% B for further 2 min. Full HR–MS experiments were performed in positive ion mode from 350 to 1600 *m*/*z* with a resolution of 120,000 (at 400 *m*/*z*). The capillary temperature was set at 275 °C, the source voltage at 1.7 kV, and the S-Lens RF level at 67%. In the data-dependent acquisition mode, the 10 most intense ions were fragmented by collision-induced dissociation (35% normalized collision energy for 10 ms, 2 *m*/*z* isolation width, activation q of 0.25). HR–MS and MS/MS data were generated by Xcalibur 3.0.63 (Thermo–Fisher Scientific, San Jose, CA, USA). MS/MS spectra were analyzed by Proteome Discoverer software (version 2.2, Thermo Fisher Scientific, San Jose, CA, USA) with the SEQUEST HT cluster search engine (University of Washington, licensed to Thermo Electron Corporation, San Jose, CA, USA) against the UniProtKB human database (reviewed, 26,806 entries, release 2023_01, available online: https://www.uniprot.org/). Proteins were identified with at least one unique peptide and the database search parameters were as follows: peptide mass tolerance 10 ppm; fragment ion mass tolerance 0.6 Da; 2 missed tryptic cleavages; false discovery rate (FDR) 0.01 (strict) and 0.05 (relaxed); carbamidomethylation of cysteine as fixed modification; oxidation of methionine and tryptophane, serine/threonine phosphorylation, N-terminal pyroglutamic residue, and N-terminal lysine acetylation as dynamic modifications. Peptides were filtered for high confidence and a minimum length of 6 amino acids. Only proteins identified in at least 30% of one group (D, S, or H) were submitted to label-free quantification (LFQ) by using the feature mapper and precursor ions quantifier nodes for the precursor quantification. To maximize the settings of the software, the MS data from all the samples were loaded in a unique comparison analysis in the Proteome Discoverer software (version 2.2), so that LFQ abundances could be determined with high confidence. LFQ quantification included all the unique peptides identified for each protein, thus at least one unique peptide, and excluded peptides which can be shared with different protein groups (razor peptides). All the LFQ abundances were normalized by the software on the total peptide amount measured in each sample. Keratins and hemoglobin were considered contaminants and thus excluded. 

The mass spectrometry proteomics data have been deposited into the ProteomeXchange Consortium via the PRIDE [14] partner repository with the dataset identifier PXD050863.

**Statistical analysis**

The abundances of 2009 proteins were automatically normalized against the total amount of tryptic peptides by Proteome Discoverer software. Data above the limit of detection (LOD) of our MS apparatus were log_2_ transformed and normalized with respect to the medians. Data below the LOD were conventionally set as log_2_(1000), a value below the minimum of the entire dataset (Supplementary File S1). The choice of this value was not critical as none of the statistical methods adopted (non-parametric tests and Random Forest) required specific data distributions.

Non-parametric Mann–Whitney (MW) and Kruskal–Wallis (KW) tests were applied to identify proteins with different abundances among the three groups of samples, S, D, and H. Even if data were paired (i.e., obtained from the same subjects), MW tests were performed in unpaired mode to evaluate the average changes among subjects, and not within subjects, which is more relevant for diagnostic considerations. Given the large number of proteins (2009) and tests (4 for each protein), FDR was controlled by the method of Benjamini–Hochberg [15]. Only tests with *p*-values < 0.05, an absolute fold change (FC) ± 1.5, and a cumulative FDR < 5% were considered significant.

Random Forest (RF) analysis [16] was used to provide a classification of the samples in the three types of samples (S, D, and H), using a subset of relevant proteins selected by the Boruta method [17]. Selection was performed by comparing the ability of proteins to discriminate different groups with that of shadow variables obtained by random permutations. Only proteins determined to be significantly more important than shadow variables were selected for RF. The RF parameters, such as the number of trees to grow and the number of proteins sampled for each split, were preliminarily tuned to optimize the classification accuracy. RF classification was validated by the “out–of–bag” samples. This method consists in using only about two-thirds of the samples for each decision tree. The classification obtained with these samples is then tested using the remaining one-third of the samples (“out–of–bag”). This procedure is repeated for each of the planned number of trees (from which the term of Random Forest comes), each time randomly selecting the samples for classification and those for validation. The overall accuracy was ultimately assessed as the average of the “out-of-bag” errors. The importance of the selected proteins for RF classification was assessed by the mean decrease accuracy score (MDA). This was computed as the decrease in classification accuracy resulting after permuting each protein. Diagrams of RF classifications were obtained by multidimensional scaling using proximity values between each pair of samples. Proximity between a pair of samples was evaluated as the normalized frequency of trees that contained the two samples in the same end node. Multidimensional scaling was computed using the singular value decomposition method, which ensures a matrix factorization that is numerically accurate even in the presence of a high degree of multicollinearity. 

Non-parametric tests and RF analysis were performed using the software R (version 4.4.1) [18].

GraphPad Prism software (version 5.0) was used to analyze correlations among LFQ abundances and clinical and demographic data. Either the Pearson or Spearman correlation test was applied based on the normality condition of data assessed by the D’Agostino and Pearson omnibus normality test. A *p*-value < 0.05 was considered accepted.

**Pathway/process enrichment analysis**

Pathway/process enrichment analysis was performed following the protocol of Reimand J. et al. [19] for defined gene lists. Briefly, two separate gene lists were analyzed: one including all the proteins identified in the present study, and one containing the proteins that were significantly varied according to the statistical analysis. g:Profiler (version e111_eg58_p18_30541362, database updated on 25 January 2024) was used with the functional profiling tool g:GOSt against the *Homo sapiens* organism with the following data sources: biological process annotations from gene ontology and the Reactome database. Electronic GO annotations were excluded, and *p*-values were subjected to Benjamini–Hochberg FDR correction. For the gene list of the 2009 identified proteins, only biological processes and pathways with a *p*-value < 0.01 were considered, while a *p*-value < 0.05 was considered for the gene list of the significantly varied proteins. The results from the pathway enrichment analysis were then visualized on Cytoscape (version 3.10.1, latest access on 21 May 2024) [20] using the “EnrichmentMap Pipeline Collection”. The platform DisGeNET (version 7.0, latest access on 31 May 2024) [21] was used to verify the correspondence of our results with respect to proteins previously linked to CRC. Briefly, CRC was searched on DisGeNET using the specific disease code C0009402 and selecting the “summary of Gene–Disease Associations” (GDA). The list of proteins deposited on the platform was then compared with the list of proteins which varied among the groups according to the statistics. The GO annotations for the molecular functions and the biological processes associated with these proteins were provided by QuickGO, available in the EMBL–EBI website (available online: https://www.ebi.ac.uk/QuickGO/annotations accessed on 22 May 2024).

**Protein–Protein interaction network and topological analysis**

Protein–protein interaction (PPI) analysis was performed on the 2009 identified proteins by Cytoscape (version 3.10.1, latest access on 1 July 2024), and STRING (version 2.0.3, latest access on 1 July 2024) based on protein queries to obtain a full network with a confidence cutoff of 0.7 against the *Homo sapiens* database. The topological parameters of the PPI network were calculated using the Network Analyser tool on Cytoscape, and the degree, betweenness centrality, and closeness centrality were utilized to evaluate the importance of the network nodes [22]. For accessing key nodes in the extended network, members were first ranked by their degree and betweenness centrality values. The top-scoring proteins, corresponding to 10% of the total number of nodes, were then selected. Key nodes close by high degrees were considered hub proteins, showing a high number of connections with other partner proteins, while nodes with high betweenness centrality, namely bottleneck proteins, were considered key connectors in the network and form the backbone network. The nodes with the highest closeness centrality values represent the topological center of the network. Among the hubs and bottlenecks, three key node subgroups were identified: (i) mixed hub–bottleneck proteins, which showed both high degrees and large betweenness centrality values; (ii) pure hub proteins, with high degrees but small betweenness centrality values; and (iii) pure bottlenecks, with large betweenness centrality values and low degrees [23].

To evaluate a possible correlation between the results of the topological analysis and those of the statistical analysis, the list of proteins whose levels were significantly varied was compared with those of key nodes in the backbone network.

## 3. Results

### 3.1. Statistical Analysis of LFQ Abundances Measured in S, D, and H Samples

#### Differential Statistical Analysis

The extensive proteomic analysis of samples from the three types of analyzed tissues yielded a total of 2009 identifications (Supplementary File S1 and ProteomeXchange entry PXD050863). The complete data, including the normalized log_2_ LFQ abundances of the 2009 identified proteins in the three groups S, D, and H, medians, and interquartile ranges, are also shown in Supplementary File S1. Naturally occurring peptides and small proteins, with an MW ranging from 5 kDa to 20 kDa, comprised 321 out of the 2009 identifications, among which 24% were identified with a unique peptide. Overall, 17% of the 2009 identifications were obtained with a unique peptide.

Ninety-one proteins showed significant differences in at least one comparison among the three groups. The results of the statistical tests (*p* values and absolute FC) are reported in Table 2. Considering the individual tests, the significantly varied proteins were as follows:

- 30 in the S vs. H comparison (MW test), with 19 less-abundant and 11 more-abundant proteins in the S tumor region;
- 31 in the D vs. H comparison (MW test), with 16 less-abundant and 15 more-abundant proteins in the D tumor region.

The proteins changing simultaneously in multiple comparisons were as follows:

- 20 in both S vs. H and D vs. H comparisons (MW test), with 18 less-abundant and 2 more-abundant proteins in both tumor regions;
- 2 in both S vs. D and D vs. H comparisons (MW test).

KW tests highlighted 64 proteins with levels that significantly varied among the S, D, and H groups. Fifty-six of significant KW tests concerned proteins already found by the MW tests. Eight proteins were significantly varied only based on the KW tests (Table 2), showing variations among the three groups and including three proteins never detected in D tumor samples, two proteins never detected in S tumor samples, and one protein (complement C4-B) never detected in either S or H samples. Finally, NADH dehydrogenase ubiquinone flavoprotein subunit 1 (NDUFV1) was not detected in any tumor sample.

For simplicity, proteins with significantly lower or higher levels in tumor samples are indicated in the text as “less-abundant” or “more-abundant”, respectively.

Overall, 61% of FCs were between 2 and 10, and 38% were higher than 100. Such high FCs were due to the lack of detection of some proteins, i.e., they were below the LOD of our apparatus. Only one protein, 182 kDa tankyrase-1-binding protein (TAB182), had an FC less than 2 between S and H samples, with S<H. 

Twenty-two percent of the proteins showed similar changes when comparing tissues from tumor regions (both S and D) and healthy colon mucosa, suggesting they may be basal hallmarks of CRC but not distinctive of the intra-tumor heterogeneity represented here by the superficial layer and the inner deep region. Among the 18 proteins with the lowest levels in tumor samples, one peptide YY (PYY), was never detected in D samples (Table 2). The two more-abundant proteins in both analyzed tumor regions were transforming growth factor-beta-induced protein ig-h3 (beta ig-h3) and guanine nucleotide binding protein G(i) subunit alpha 2 (Galphai2). 

Sixty percent of the proteins showed significant changes either in S or in D tumor samples with respect to the H samples, while their levels were not significantly different between the two tumor regions. Notably, among the less-abundant proteins in S vs. H and D vs. H comparisons, eight were never detected in S, and one, synaptogyrin-2, was never detected in D tumor samples (Table 2). Among the more-abundant proteins in S vs. H and D vs. H comparisons, six proteins were never detected in H control samples, and one, transmembrane protein 263, was never detected in either S or H samples (Table 2). The variations were significant in only one of the tumor regions, even if not strong enough to distinguish S from D samples by means of the differential statistical analysis, and appeared as signals of divergence, which was demonstrated by the RF classification analysis described in the next paragraph.

Only two proteins showed significant differences not only in the comparison with H control samples, but also between the S and D tumor regions: GRIP1-associated protein 1 (GRASP-1), with the highest levels in D samples and never detected in S samples; and sorting nexin-18, with the highest levels in S samples and never found in D samples. 

The gene ontology (GO) ID annotations for the molecular function and biological processes related to these 91 proteins with statistically significant variations are reported in Table S1 (Supplementary File S2). Moreover, to obtain information about the association among proteins of interest and previous literature focused on CRC protein biomarkers, we tested the 91 proteins with the DisGeNET tool, and the resulting DGA scores are indicated in Table 2. About 55% of the 91 varied proteins were already associated with CRC in previous investigations based on DisGeNET results. Table 2 also reports references concerning the association of these proteins and CRC found through classical bibliographic research, which in some cases accomplishes the DisGeNET results. The techniques used for protein identification in previous studies are indicated in the last column of Table 2. As far as we know, 23 of the 91 proteins have never been associated with CRC before this study.

### 3.2. RF Classification Analysis

**Figure 1:**
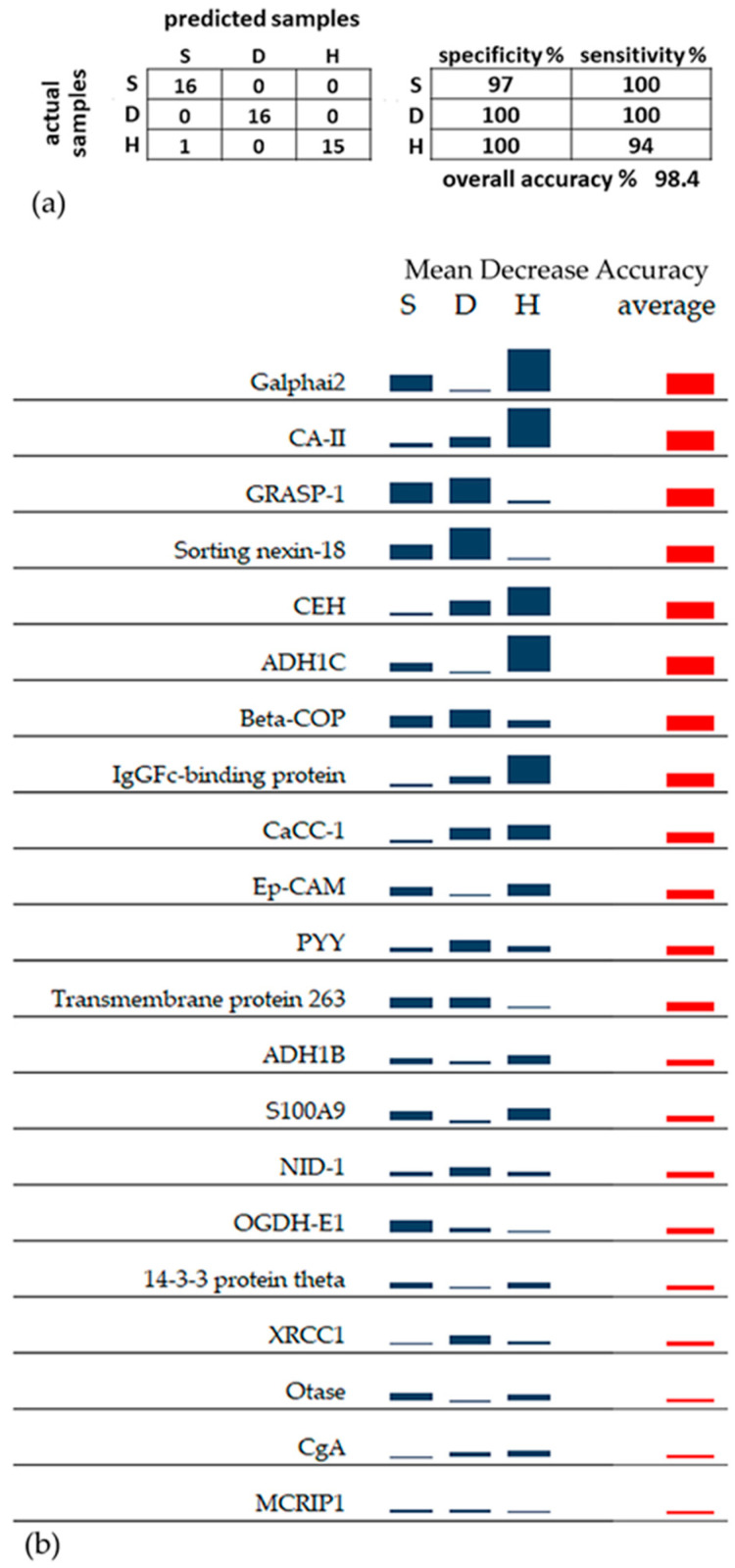
(**a**) Confusion matrix and sensitivity/specificity of RF classification. (**b**) Sparkline graphic representing the relative importance of the 21 proteins selected for RF classification of the S, D, and H groups, calculated as MDA. Proteins with a higher average MDA have a greater importance in the RF model. Blue columns show the MDA scores calculated for each group. The red column shows the average MDA score.

RF classification was performed using 21 proteins selected by the Boruta method among the entire dataset of 2009 identified proteins. Interestingly, the 21 proteins selected for RF (highlighted in gray in Table 2) were all included among the 91 proteins with significant changes identified by the MW and KW tests. RF analysis correctly classified almost all samples using the 21 selected proteins, with an overall accuracy of 98.4% (Figure 1a). No errors were found in the classification of S and D tumor samples. The only misclassification occurred with a sample from the H group that was attributed to the S group. The different contributions of the 21 proteins to this highly accurate RF classification are expressed by the MDA score. The MDA score evaluates the decrease in classification accuracy after randomly permuting (i.e., scrambling) the LFQ abundance values of a single protein among the three groups, S, D, and H: the greater the decrease in classification accuracy after permuting a certain protein, the greater the importance, or MDA score, of that protein. Figure 1b shows the MDA scores of the 21 proteins calculated for each group, S, D and H, (blue columns), and the average MDA score (red column). The corresponding numerical values of MDA scores are reported in Table S2 (Supplementary File S2). Based on the average MDA score, Galphai2, carbonic anhydrase 2 (CA-II), GRASP-1, sorting nexin-18, bifunctional/cytosolic epoxide hydrolase 2 (CEH), alcohol dehydrogenase 1C (ADH1C), coatomer subunit beta (beta-COP), and the protein binding the Fc portion of IgG (IgGFc-binding protein) appeared to be the most important proteins for the classification of the three types of analyzed tissues. All these eight proteins achieved an average score > 0.020 (Table S2). Based on the MDA scores of the single groups, Galphai2, CA-II, ADH1C, CEH, and IgGFc-binding protein, showing the highest MDA score in the H sample group, appeared to classify the non-tumor control tissue well. GRASP-1 was a good classifier for both superficial and deep regions of the tumor, showing the highest MDA scores in both the S and D sample groups. Meanwhile, sorting nexin-18 and beta-COP, with the highest MDA score in the D sample group, more accurately classified the deep tumor region.

**Figure 2:**
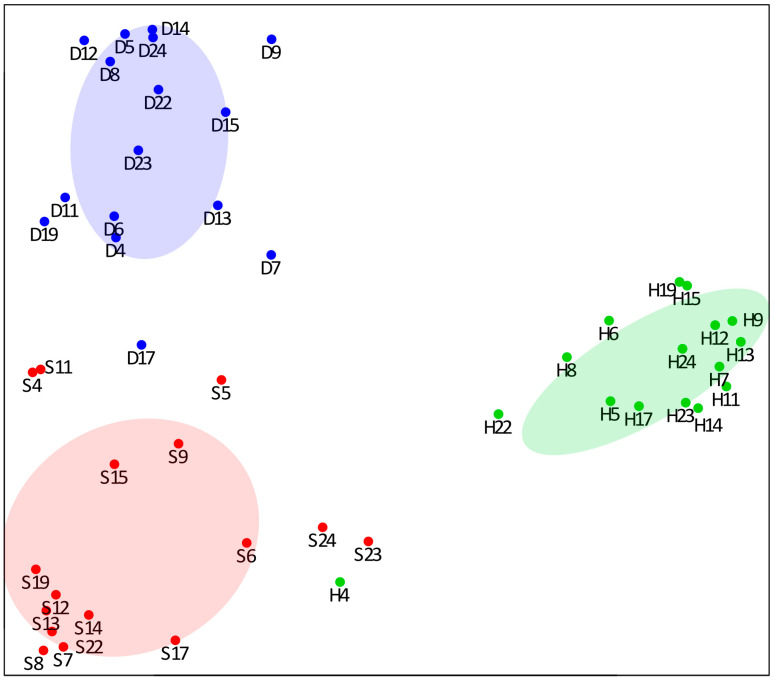
Multidimensional scaling plot showing the relationships among the three groups of samples (S, red; D, blue; H, green), using the proximity values calculated by RF. Patient identification numbers correspond to those shown in Table 1. Each group is delimited by a dispersion ellipse with a confidence of 1.6 standard deviations.

A 2D representation of RF classification is shown by the multidimensional scaling plot obtained from the proximities among samples (Figure 2). The separation of the three groups of samples is strongly evident. All the samples from healthy mucosa are very detached from the S and D tumor samples, with the only exception of the H4 sample, also resulting from a confusion matrix. The perfect discrimination between the internal and the peripheral region of the tumor emphasizes different proteomic profiles that could not be predicted due to the few significant differences between these two groups obtained by single univariate tests.

**Figure 3:**
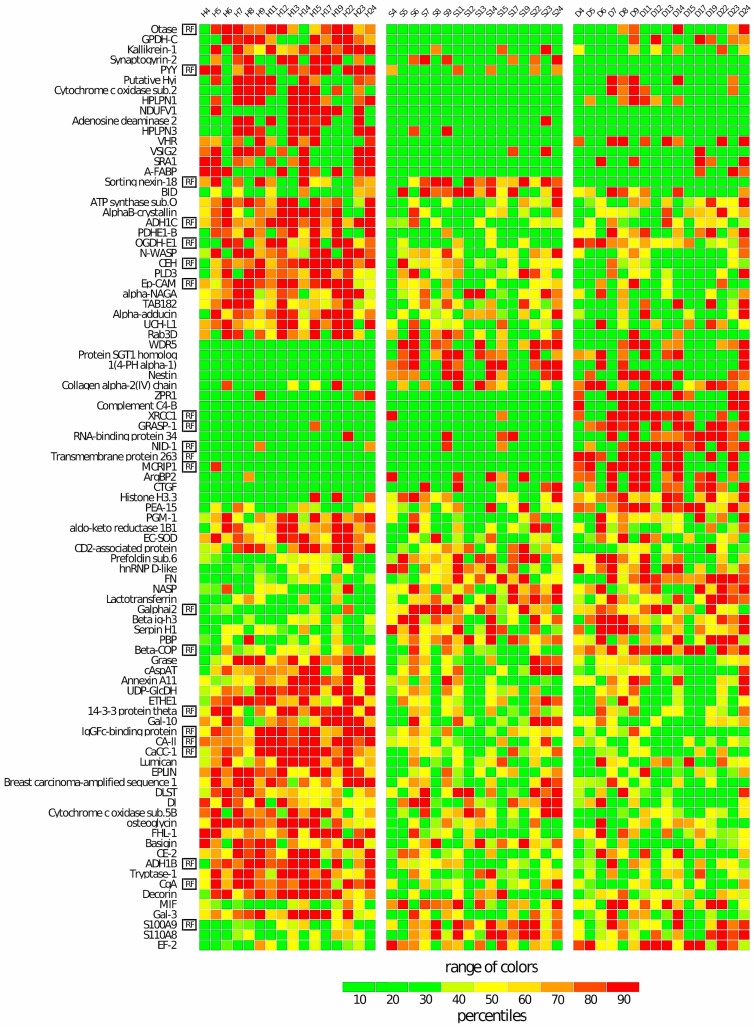
Heatmap of the 91 proteins showing significant changes among the three groups of samples. Each row of the heatmap represents a specific protein, and each column represents a single subject. The “RF” labels indicate the 21 proteins used for RF classification. For each protein, the color of each cell reflects the percentile value of each subject relative to all 48 subjects present in the same row. Shades of green and red represent decreasing and increasing percentiles, respectively, from the median. To obtain homogeneous blocks of color, the proteins were preliminarily ordered vertically based on the output of a hierarchical cluster analysis. Note that the 21 RF proteins are spread across all the major clusters produced by the 91 differentially expressed proteins. This suggests that the RF proteins span the full range of the different relationships among the proteins found to be varied among the three groups.

The significant differences in the LFQ abundances of the 91 proteins among the three groups are also evident in the heatmap of Figure 3. In addition, the heatmap allows for the identification of the differential pattern of the 21 proteins (marked as “RF” in the first column of the figure) used for the RF classification, which were spread across all the major clusters produced by the 91 proteins. Overall, it is interesting to underline a major homogeneity in the protein profiles of the H control samples compared to the two tumor regions, S and D, which, instead, were shown to be more heterogeneous among individuals.

### 3.3. Enrichment Analysis of Biological Processes and Pathways

**Figure 4:**
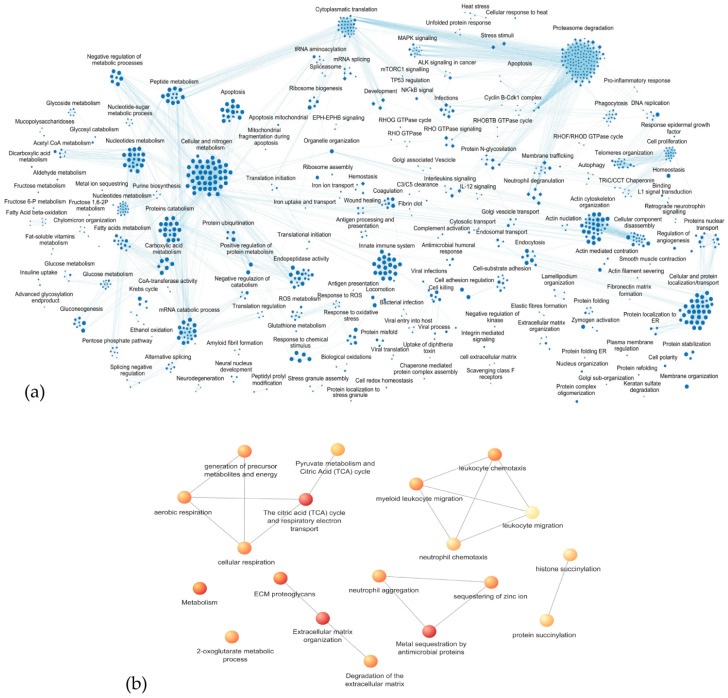
Enrichment analysis via g:Profiler coupled with Cytoscape EnrichmentMap Pipeline Collection of the following: (**a**) of all the 2009 proteins identified in the study, only functional networks defined by the processes with *p*-values < 0.01 are shown; (**b**) the 91 proteins showing significant variations in the statistical differential analysis. Colors refer to FDR, from the least significant (light yellow, FDR 0.04) to the most significant (red, FDR 0.002).

An enrichment analysis was first performed on all the 2009 identified proteins, highlighting a predominant involvement in biological processes and pathways related to metabolism (both synthesis and catabolic pathways) and in its regulation, especially regarding proteins, fatty acids, and nucleic acids. Furthermore, highly significant FDR values were obtained for biological processes/pathways included in the innate immune system, response to infections and oxidative stress, apoptosis, proteasome activity, actin cytoskeleton organization and assembly, cellular and protein localization, transport, and membrane trafficking. Figure 4a shows the functional network defined by processes with *p*-values < 0.01. Supplementary File S3 reports the GO/Reactome entries, FDR, and proteins associated with each process.

Considering the 91 proteins showing significant variations in the statistical differential analysis, the enrichment analysis identified the networks of biological processes (GO) or pathways (Reactome) with an FDR < 0.05, as described in Table 3 and shown in Figure 4b.

#### 3.3.1. Proteoglycans and Extracellular Matrix Dynamics

In this first cluster of biological processes/pathways, decorin, lumican, collagen alpha-2(IV) chain, and fibronectin (FN) were identified with significant FDR, forming a network of three processes: (i) degradation of the extracellular matrix (ECM), also involving mimecan (or osteoglycin), basigin, nidogen-1 (NID-1), and tryptase alpha/beta 1 (tryptase-1); (ii) proteoglycan dynamics in the ECM, involving hyaluronan proteoglycan link protein 1 (HPLPN1); (iii) ECM organization also involving HPLPN1, basigin, NID-1, tryptase-1, serpin H1, and prolyl 4-hydroxylase subunit alpha-1 (4-PH alpha-1) (Figure 4, Table 3). The process identified with the lowest FDR, among the three, was the ECM organization involving several less-abundant proteins, such as decorin and tryptase-1, with the lowest levels in both tumor regions, basigin in the deep part of the tumor, and lumican, mimecan, and HPLPN1 on the surface of the tumor (Table 2). HPLPN1 was never detected in S samples. FN, collagen alpha-2(IV) chain, serpin H1, and NID-1 were found to be, instead, more-abundant proteins in D tumor samples, while 4-PH alpha-1 was more abundant in S samples and not detected in healthy control samples.

As reported in Supplementary Table S1, the GO annotation ID for molecular function GO:0030021/30020 “extracellular matrix structural constituent conferring compression resistance/tensile strength” was associated with decorin, lumican, collagen alpha-2(IV) chain, mimecan, and HPLPN1. The ID annotation for the “collagen fibril organization” process (GO:0030199) was associated with lumican, serpin H1, and 4-PH alpha-1, the latter of which directly modifies collagen molecules with its enzymatic “procollagen–proline 4-dioxygenase activity” (GO:0004656). Collagen alpha-2(IVA) chain was associated with the processes of “collagen-activated tyrosine kinase receptor signaling pathway” (GO:0038063), and angiogenesis (GO:0001525). FN is an integrin interactor (GO:0005178). FN and HPLPN1 are associated with ID for the process GO:0007155 “cell adhesion”, and FN and NID-1 are extracellular matrix structural constituents (GO:0005201) and are associated with the ID for process GO:0007160 “cell–matrix adhesion”. Basigin is associated with several IDs for process, among them ID GO:1904466 “positive regulation of matrix metallopeptidase secretion” is well in accordance with the organization and degradation of ECM, as found here. Analogously, tryptase-1 is associated with the process “extracellular matrix disassembly” (GO:0022617). Based on their GO annotations (Supplementary File S2, Table S1), several of these proteins are integrin interactors (GO:0005178). The building and remodeling of ECM, including the existence of a protein network essential for maintaining the homeostasis of physical and functional connections in the ECM, require the activity of integrins. We detected integrins alpha-V and alpha-M (Supplementary File S1) but, unlike their interactors, integrins did not show significant variations among the compared groups.

#### 3.3.2. Metabolic Processes

Eight GO/Reactome entries identified metabolic processes (Table 3, Figure 4), predominantly concerning the citric acid cycle, the oxidative phosphorylation (OXOPHOS) processes, and overall mitochondrial processes generating energy. Galphai2 was the only protein in this cluster of functional networks showing the highest abundance in both S and D tumor samples. Galphai2 is a component of several signaling pathways regulating the metabolic processes, with its principal association being the GO annotation GO:0007165, “signal transduction” (Supplementary File S2, Table S1). All the other proteins were less abundant in the tumor samples. Among them, ATP synthase subunit O had the lowest level in both D and S samples, and NDUFV1 was not detected in any tumor samples (Table 2). Pyruvate dehydrogenase E1 subunit beta (PDHE1-B), 2-oxoglutarate dehydrogenase E1 (OGDH-E1), and cytochrome c oxidase subunit 2 were less-abundant proteins in the S tumor samples (Table 2). OGDH subunit E2, namely dihydrolipoyllysine-residue succinyltransferase (DLST), cytochrome c oxidase subunit 5B (Complex IV), and persulfide dioxygenase ETHE1 were less-abundant proteins in D tumor samples (Table 2).

The ID GO annotations for the molecular functions and specific processes of these proteins are indicated in Table S1 (Supplementary File S2). NDUFV1 is a component of Complex I of the electron transport chain (GO:0006120), while cytochrome c oxidase subunit 2 and subunit 5B are components of Complex IV (GO:0006123). OGDH-E1 and DLST are components of the OGDH complex that converts 2-oxoglutarate to succinyl-CoA in the Krebs cycle (GO:0006103), while PDHE1-B is a component of the PDH complex that synthesizes acetyl-CoA from pyruvate (GO:0006086). ETHE1 is an enzyme important for mitochondrial redox homeostasis and participates in the “glutathione metabolic process” (GO:0006749). Several proteins participate in lipid metabolism, namely A-FABP and cytoplasmic glycerol-3-phosphate dehydrogenase (GPDH-C), which were never detected in S samples; aldo-keto reductase 1B1 was found to be a less-abundant protein in D tumor samples; CEH and carboxylesterase 2/cocaine esterase (CE-2) showed the lowest level in both S and D tumor samples (Table 2). As reported in Table S1 (Supplementary File S2), CEH, CE-2, and aldo-keto reductase 1B1 are associated with the ID GO:0006629 “lipid metabolic process” and GO:0006693 “prostaglandin metabolic process”. CEH, also known as EPHX2, is also involved in “cholesterol homeostasis” (GO:0042632). CEH is a cytosolic bifunctional enzyme with lipid epoxide hydrolase and lipid phosphatase activities, and it principally metabolizes xenobiotics and cytotoxic epoxides derived from fatty acids. A-FABP is a long-chain fatty acid transporter (GO:0015909), and GPDH-C is involved in the glycerol-3-phosphate metabolic process (GO:0006072). Aldo-keto reductase 1B1 is also involved in the “fructose biosynthetic process” (GO:0046370) and “carbohydrate metabolic process” (GO:0005975). It catalyzes the NADPH-consuming reduction of glucose to sorbitol, and the next conversion to fructose in the polyol pathway.

Phosphoglucomutase-1 (PGM-1) was found to be a less-abundant protein on the surface of the tumor. It is noteworthy that the PGM-1 proteoform identified in this study was phosphorylated at Ser369, a PTM already reported in the human databank UniProt-KB, and acetylated at Lys370, a novel PTM for this enzyme. The PTMs identified in this study are reported in Supplementary File S1. PGM-1 is directly involved in the “glucose metabolic process” (GO:0006006). This functional cluster of proteins was also identified by proteins involved in proteoglycan metabolism: in addition to lumican, decorin, and mimecan, UDP-glucose 6-dehydrogenase (UDP-GlcDH) was also identified, with the lowest level in the tumor (Table 2). UDP-GlcDH is involved in glycoprotein and proteoglycan biosynthesis, as indicated by the association with the GO ID annotations “heparan sulfate proteoglycan biosynthetic process” (GO:0015012) and “chondroitin sulfate biosynthetic process” (GO:0030206). All-trans-retinol dehydrogenase NAD^+^ (ADH1B), ADH1C, and CA-II showed the lowest levels in both S and D tumor samples (Table 2). Alcohol dehydrogenases 1B and 1C (ADH1B and ADH1C) are enzymes principally involved in the “alcohol metabolic process” (GO:0006066), while CA-II regulates intracellular pH homeostasis (GO:0051453) (Supplementary File S1, Table S1).

Cytoplasmic aspartate aminotransferase (cAspAT), neural Wiskott–Aldrich syndrome protein (N-WASP), and 5′–3′ exonuclease phospholipase D3 (PLD3) were identified as less-abundant proteins in the deep part of the tumor (Table 2). cAspAT is associated with several GO annotations for biological processes, including GO:0006532 “aspartate biosynthetic process”, GO:0006094 “gluconeogenesis”, and GO:0006103 “2-oxoglutarate metabolic process”. N-WASP is involved in “actin cytoskeleton organization” (GO:0030036), while PLD3 hydrolyzes phosphatidylcholine to generate phosphatidic acid (GO:0004630, molecular function), and it is associated with the ID annotation “DNA metabolic process” (GO:0006259) (Supplementary File S1, Table S1).

Finally, basigin was included in this functional cluster due to its multifunctional activity as a membrane protein regulating different processes (Table S1, Supplementary File S2).

#### 3.3.3. Leukocyte Migration and Aggregation

Another interesting functional network concerned the “migration and chemotaxis of neutrophils and myeloid leukocytes” (Figure 4, Table 3) and involved proteins more abundant in S tumor samples, such as S100A9, S100A8, and platelet basic protein (PBP). It also included more-abundant proteins in the D tumor samples, such as macrophage migration inhibitory factor (MIF). In addition, proteins less abundant in the tumor, such as basigin, N-WASP, Galectin-3 (Gal-3), and chromogranin-A (CgA), were identified in this functional network. Gal-3 and CgA showed the lowest levels in both tumor regions. Moreover, the S100A9 and S100A8 proteins, together with lactotransferrin, were identified with a highly significant FDR in the process of “metal sequestration by antimicrobial proteins”, which was linked to “neutrophil aggregation” and “zinc ion sequestration” (Figure 4, Table 3). As evidenced by the GO annotations reported in Table S1 (Supplementary File S2), S100A8 and S100A9 show several associations with the dynamics of the innate immunity and the inflammatory response (GO:0006954, GO:0061844), as well as PBP. MIF exhibits “cytokine activity” (GO:0005125) and is linked to several signaling pathways, similarly to basigin. PBP is a potent chemoattractant and activator of neutrophils (GO:0006935). Lactotransferrin plays a role as a defense protein (GO:0031640). The regulatory activity on the cytoskeleton carried out by N-WASP is fundamental for the motility of leukocytes. CgA and Gal-3 are associated with GO annotations concerning the chemotaxis of immune cells, including GO:0002551 “mast cell chemotaxis”, GO:0002548 “monocyte chemotaxis”, and GO:0030593 “neutrophil chemotaxis”.

### 3.4. PPI Network and Topological Analysis

The examination of the topological features of the PPI network, such as the node degree, betweenness centrality, and closeness centrality, highlighted that nodes with high degrees were identified as hub proteins. Because of their high number of interacting partners, these nodes can be considered key nodes. On the other hand, proteins with large betweenness centrality values were also identified as key nodes, known as bottlenecks, due to their central role in facilitating communication and information flow between different parts of the network. The extended network of identified proteins was composed of 1967 nodes, interacting with 20,967 edges. Proteins in the top 10% for degree values (min 59, max 200) were considered hub proteins (n = 197), and proteins in the top 10% for betweenness centrality values (min 0.003, max 0.08) were considered bottleneck proteins (n = 196). Bottlenecks constitute the backbone network consisting of 196 nodes interconnected by 1787 edges; the size of each node corresponds with the betweenness centrality values (Figure S1 in Supplementary File S2). Glyceraldehyde-3-phosphate dehydrogenase (GAPDH in the Figure S1), with the highest closeness centrality, betweenness centrality, and degree values, represented the topological center of the backbone network, allowing the flow of information among its portions. Heat shock protein 90-alpha (HSP90AA1) and actin (ACTB) assumed important positions within the backbone network, second and third, respectively, for their high closeness centrality and high values of betweenness centrality and degrees. FN and elongation factor 2 (EF-2) belong to the group of mixed hub–bottleneck proteins, as they have both high degrees and large betweenness centrality values. They showed varied levels according to the statistical analysis, and their topological parameters in the backbone network are reported in Table 4. Some proteins, showing large betweenness centrality values but low degrees (<59), were considered pure bottlenecks: beta-COP, dipeptidyl peptidase 4 (ADABP), cAspAT, Immunoglobulin heavy variable 3–43D, Gal-3, lactotransferrin, PDHE1-B, N-WASP, and WD repeat-containing protein 5 (WDR5). These proteins are indicated with an asterisk in Table 4.

### 3.5. Correlation between Proteomic and Clinical Data

**Figure 5:**
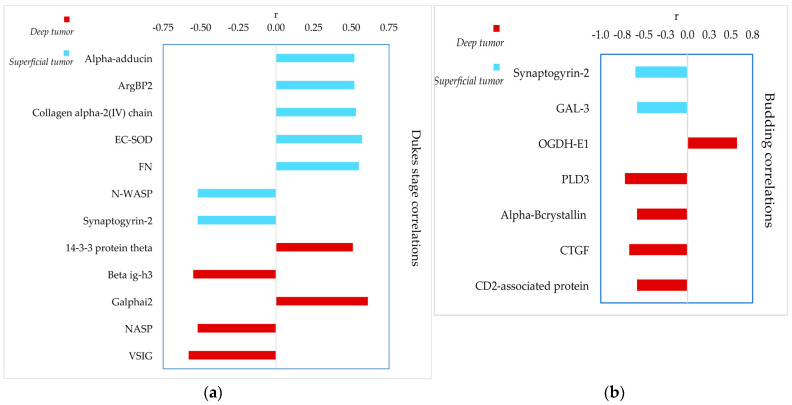
Results from correlation analysis: (**a**) proteins correlated with the Dukes stage and (**b**) with the budding index in the two tumor tissues; D in red, S in cyan; positive and negative r coefficients are reported in the plot.

The LFQ abundances of the 91 proteins showing variations in relation to the intra-tumor localization and to the healthy tissue were also analyzed to verify possible correlations with stratification of the patients according to the Dukes classification (A, B, and C stages), and to the budding index, ranging from 0 to 3. Figure 5 shows the *r* values of the significant positive and negative correlations with the Dukes stage (Figure 5a), and budding index (Figure 5b). All the results are reported in Supplementary Table S2 with the *p*-values and *r* coefficients.

In the superficial region of the tumor, significant positive correlations with the worsening of the Dukes stage, from A to C, were observed for alpha-adducin, sorbin-SH3 domain-containing protein 2 (also known as Arg-binding protein 2, ArgBP2), collagen alpha-2(IV) chain, extracellular superoxide dismutase (EC-SOD) and FN, while negative correlations were found for N-WASP and synaptogyrin-2. In the deep region of the tumor, 14-3-3 protein theta and Galphai2 correlated positively with the advancement of the Dukes stage, while beta ig-h3, nuclear autoantigenic sperm protein (NASP), and V-set-immunoglobulin domain-containing protein 2 (VSIG2) exhibited negative correlation.

Significant correlations were also found with the budding index. Synaptogyrin-2 and Gal-3 abundances decreased in superficial tumor regions as the budding index increased. In the deep tumor region, a similar correlation was observed for PLD3, alphaB-crystallin, CCN family member 2 or connective tissue growth factor (CTGF), and CD2-associated protein, while OGDH-E1 showed an opposite trend.

## 4. Discussion

Proteomic data obtained from colorectal carcinoma samples were analyzed using a machine-learning approach for the evaluation of different aspects: (i) significant quantitative variations in the proteins among the compared groups to evidence less- or more-abundant proteins in different regions of the tumor, superficial and deep, and with respect to the distant healthy colon tissue from the same patients; (ii) identification of sensitive and specific proteins capable of classifying S and D tumor regions and H samples; (iii) significant correlations between protein abundances and diagnostic clinical parameters, such as the Dukes stage and budding index; (iv) identification of biological processes associated with the proteins that differentiate and categorize tumor and healthy samples; (v) identification of key proteins acting as central nodes in PPI networks associated with the tumor. It is worth noting that CRC budding growth and the budding index are associated with EMT. Indeed, EMT is evidenced by alterations in the tissue architecture at the deep invasive tumor margins, referred as “budding margins”, which are infiltrative margins with solid cell nests formed by one to five cancer cells that acquire motility and infiltrate the peritumor connective tissue [13].

Our study found a panel of proteins that, based on these considerations, appeared to have a diagnostic potential and were shown to be of interest for further targeted investigations devoted to the data validation with an immunological independent approach. As expected, given the great intra- and extra-tumor heterogeneity already documented for CRC [2,3], the two parts of the tumor were clearly distinguishable at the proteomic level from each other and from the non-tumor samples, despite coming from the same individual. Moreover, several proteins were varied only in one of the two tumor regions, and, in several cases, proteins were never detected in either the superficial or deep tumor region. The divergence in the protein profiles between the superficial and deep tumor regions, suggested by MW and KW comparison tests, was confirmed by the RF classification analysis. Consequently, the same biological process was down- or up-regulated in the two different regions of the tumor. This suggested that the two parts of the tumor employ different metabolic, signaling, and regulatory strategies and adapt differently to the environment in which CRC cells differentiate and proliferate. The superficial region of the CRC is the original area from which the tumor grows inwards and expands into healthy tissue, it is the area in contact with the intestinal lumen and the gut microbiota. The deep region is the part of the tumor that infiltrates and invades the healthy tissue layers of the intestinal wall, where the budding of cancerous cells develops and from which the metastases mature and detach. CRC cells, to ensure their survival, must be capable of controlling the TME, modifying the ECM, initiating EMT, altering the metabolism of neighboring cells and differentiating them, as well as transforming the locally recruited immune cells. All processes were individuated in association with CRC by this proteomic study. It should be emphasized that this study highlighted quantitative and qualitative variations in protein profiles among the three types of compared tissues, variations that may be associated with differential gene expression or protein clearance rather than the regulation of protein activity.

### 4.1. Quantitative Differences in Protein Profiles in Tumor and Healthy Tissues and the Implication of Biological Processes/Pathways

Ninety-one proteins with significantly different abundances among tumor and healthy samples were identified. The predominant changes concerned 60% of varied proteins, which were less abundant in either superficial or deep tumor tissue, or in both regions compared with non-tumor control samples. Only about 31% of the varied proteins were found to be more abundant in the tumor tissue. The quantitative protein changes determined in tumor and healthy tissues reflect the biological processes and pathways in which these proteins are involved. It was evident that many of the identified proteins are implicated in various type of tumors, where their roles and activity regulation may change depending on the specific microenvironment of the tumor. In addition, several processes appeared to be affected by different regulatory proteins showing opposite variation trends in the two analyzed areas of the tumor, suggesting very complex networks of possible protein interactions distinctly utilized by the two types of CRC cells. Finally, the results of the topological analysis emphasized the findings obtained from the statistical comparison and RF-classification, confirming the biological significance of some proteins in the context of CRC. The analysis of PPI networks identified a complex backbone network consisting of 196 nodes, in which some proteins, showing significant variations by statistical comparison, were central nodes, such as FN and EF-2. These proteins have constrained evolution and crucial roles for cell life, mediating numerous interaction paths and protein connections’ flow. These properties are crucial in the context of CRC; for instance, FN can bind signaling receptors in the ECM, and it can stimulate CRC cell proliferation and differentiation by participating in several PPI networks [56]. The data found on FN and EF-2 suggested that their down- or up-regulation can affect the different biological pathways that flow through FN and EF-2, and this may be of interest for studying the multifaceted carcinogenesis and progression of CRC. Among the pure bottleneck proteins, beta-COP, cAspAT, Gal-3, lactotransferrin, PDHE1-B, N-WASP, and WDR5 resulted in significantly varied tumor samples based on statistical comparisons. Beta-COP was also found as a classifying component by RF analysis. These proteins, based on topological analysis, can interact with other key nodes of the PPI network and connect co-expressed proteins. Moreover, as pure bottleneck proteins, they may be proposed as potential drug targets. In addition, Gal-3, N-WASP, and FN, were shown to correlate with the main diagnostic outcomes, such as the Dukes stage and budding index.

#### 4.1.1. Proteins Implicated in Processes Related to Proteoglycans and ECM

The findings concerning this protein cluster were suggestive for a decreased robustness of the ECM in CRC. It is well known that oncogene activation destabilizes cell–cell adhesions and stimulates the remodeling and stiffening of the ECM to reach a physical equilibrium with its microenvironment. The disruption of tissue architecture can, in turn, promote malignant transformation and tumor progression [27]. Indeed, we found a cluster of proteins typically involved in the organization, disassembly, and remodeling of ECM that showed significant variations in tumor compared to healthy control samples. Decorin, lumican, and mimecan/osteoglycin are extracellular proteoglycans that regulate collagen fiber assembly, mechanical properties of the tissue, mechano-signaling, and ECM robustness. Proteoglycans are considered tumor-suppressor and anti-metastatic agents [86], as supported by the GO annotations indicating the negative regulation of endothelial cell migration and angiogenesis. However, their expression is cancer type- and tumor stage-specific [86]. Our results were in accordance with previous studies: decorin was found to decrease in the ECM of primary CRC [75], as was osteoglycin, which can inhibit the transcription of hypoxia inducible factor-1α (HIF-1α) and vascular endothelial growth factor (VEGF) in CRC cells [33]. The alteration of ECM organization was also demonstrated by other proteins found to be less abundant in the tumor samples, namely basigin, tryptase-1, and HPLPN1. It is interesting to note that hyaluronate and proteoglycan link proteins HPLPN1 and HPLPN3, also identified in this study, are part of a unique “link module superfamily”, together with proteoglycans/hyaluronate receptors, such as Cluster of Differentiation 44 (CD44) and endothelial growth factor receptor (EGFR), and other proteins that aggregate with proteoglycans [87]. Notably, CD44 is important for cell adhesion, colonization, and priming of the metastatic niche in CRC [88], and it was suggested as a novel prognostic marker and potential therapeutic target for metastatic CRC patients [89]. The isoform CD44v6 is considered responsible for the formation of metastatic lesions in the liver and lung. CD44 (UniProt code P16070, Supplementary File S1) did not show any significant difference among the three compared groups. Basigin, also known as CD147 and a strong interactor of CD44, is a transmembrane glycoprotein with pleiotropic functions and a crucial modulating role in the transport of nutrients, migration of inflammatory leukocytes, and induction of matrix metalloproteinases [90]. The strong complex basigin–CD44, formed in the cell membrane, is a signaling membrane receptor (GO:0007166) for various protein partners, including nutrient transporters, EGFR, S100A9, and GaL-3. It has been demonstrated that the enhanced expression of basigin triggers the formation of a lipid raft-associated supramolecular complex basigin–CD44–EGFR, which appears to favor the invasive properties of tumor cells. The basigin–CD44–EGFR complex can induce the differentiation of metastatic cells through the basigin positive regulation of matrix metallopeptidase secretion [90]. It was recently found that basigin is highly expressed in serum exosomes from CRC patients [45]. Conversely to what was expected from the tumor-promoting action of basigin, we observed a down-regulation/expression of basigin in the deep region of the tumor, which appeared associated with variations in interactors different from CD44, such as GaL-3, identified as a less-abundant protein in the deep area of the tumor.

Tryptase is one of the most powerful angiogenic mediators released by human mast cells participating in the degradation of ECM components. Different mast cell populations may regulate the homeostasis of the intestinal barrier and the responsiveness during infections. Mast cell proteases can either exert pro-tumorigenic or anti-tumorigenic functions, and thus their contribution to invasion and metastasis [91]. The lowest levels of tryptase-1 in our tumor samples suggested a probable tumor-suppressing effect that has never been proposed before. It would be interesting to investigate in a future study the distributions of the mast cell populations in different regions and in the TME in CRC patients, as well as to evaluate tryptase-1 levels with immune-detection techniques complementary to the MS approach.

The identified integrin interactors, FN, collagen alpha-2(IV) chain (constituent of collagen type I), serpin H1, NID-1, and 4-PH alpha-1 protein, were found to be more abundant in the deep tumor samples. The proteomic study of Torres and coll., investigating the secretion of cancer-associated fibroblasts (CAFs) isolated from the colon tissue of a mouse model, demonstrated that CAFs, which intensely proliferate in the tumor stroma, can release FN, NID-1, collagen alpha2(IV) chain, other ECM components, and ECM-degrading proteases, to support growth, the invasion of the tumor, angiogenesis, and pro-fibrotic processes [37]. It is amazing to hypothesize that several of the ECM components and modulators identified in our samples were originated by CAFs rather than CRC cells. Moreover, NID-1 is directly involved in the paracrine induction of EMT and cell migration in CRC [61]. The positive correlation of FN and collagen alpha-2(IV) with the progression of Dukes stage found in the tumor surface was in accordance with their recognized prognostic power in CRC [37]. Interestingly, the variation in FN in our samples was the opposite to that of CD2-associated protein, a scaffold/adaptor component of molecular hubs in signaling pathways. CD2-associated protein was found to be less abundant in the deep part of the tumor, consistent with a study demonstrating the increased FN expression in silenced cells for CD2-associated protein, and the capacity of this protein to regulate migration and EMT-related pathways in CRC cells [47]. In this context, the finding of CTGF, an integrin interactor with key roles in ECM dynamics, such as the regulation of fibroblast growth and cell differentiation, is notable. CTGF was a more-abundant protein in deep tumor tissue like the other integrin-interactors, and in accordance with a recent study that reveals its tumor-promoting action in CRC [57]. CD2-associated protein and CTGF, despite exhibiting opposite variations in the deep part of the tumor, showed a similar negative correlation with the budding index, with higher abundances in patients with lower budding.

Another interesting protein in this context was alphaB-crystallin, a molecular chaperone promoting cell survival and preventing apoptosis, as indicated by GO annotations. An in vitro study observed that alphaB-crystallin can promote the invasion and metastasis of CRC cells through EMT processes, with a molecular mechanism negatively affecting the expression of the epithelial cell adhesion marker E-caderin and positively affecting the expression of mesenchymal markers such as vimentin and FN [25]. Despite these tumor-promoting effects, alphaB-crystallin was a less-abundant protein in the superficial tumor samples and in the deeper region its abundancy was significantly lower in patients with higher budding index, consistent with the recent study of Pagano et al. [27]. Although the enrichment analysis did not include beta ig-h3 among the proteins identifying the ECM dynamics, it is an ECM constituent that interacts with integrins and inhibits cell adhesion (Table S1). Beta ig-h3, found with the highest abundance in both regions of the tumor, has been proposed as a marker of metastatic primary colon carcinomas, and its pro-tumorigenic activity was found more relevant in the less severe Dukes stages [81], in accordance with our results highlighting that the beta ig-h3 abundance in the deep tumor was lower in patients with an advanced Dukes stage.

All these data point out both the significance of the ECM as a structural scaffold in the tumor stroma of CRC and the ECM participation in the TME affecting CRC cells’ behavior and metastatic potential. ECM composition has been linked to patient outcome and the response to surgical and oncological therapy in CRC patients, suggesting that ECM may be a valuable area for developing novel biomarkers and targeted therapy [75]. The activity of ECM factors is strictly associated with that of secreted components implicated in the innate immunity and in inflammatory processes. It has been proposed that the transition from the chronic intestinal inflammation to CRC involves changes in ECM matrix proteins, such as FN, matrix regulators like serpin H1, and matrix enzymes and inflammatory factors, such as S100A8 and S100A9 [92].

#### 4.1.2. Proteins Implicated in Leukocyte Migration and Aggregation

The impairment of processes and pathways concerning the migration and chemotaxis of myeloid leukocytes was suggested by quantitative variations in CRC tissues of proteins that are involved in the creation of an immune microenvironment able to suppress the immune response and to favor the survival of tumor cells. S100A9 and S100A8 are the principal components of this functional cluster of proteins. Our results highlighted S100A9 and S100A8 as more-abundant proteins in the superficial tumor samples, in accordance with previous studies [41]. They were both found to be up-regulated in tumor-infiltrated myeloid cells that in the TME may differentiate into “S100A8/9-expressing myeloid-derived suppressor cells” or M2 macrophages. S100A9 was indicated as a potential marker of CRC because myeloid-derived suppressor cells recruited in the TME can secrete high amount of exosomal S100A9 under hypoxic conditions [93]. S100A8 and S100A9 participate in the regulation of the TME affecting tumor genesis, progression, and metastasis through various pathways [93,94]. Moreover, they can suppress T-cell responses to weaken the tumor-killing effect [93,94]. An abolishment of the immune responses against cancer cells was also sustained by results on VSIG2, a transmembrane protein that ensures tumor immune surveillance and was found to be down-regulated in CRC [85]. Accordingly, the decreased abundance of VSIG2 in the deep part of the tumor significantly correlated with the worsening of the Dukes stage.

Based on our results, PBP and MIF, which could affect the immune microenvironment of the tumor in different regions, may be also considered CRC tumor-promoters: PBP as a growth factor stimulating the chemotaxis and aggregation of neutrophils in the superficial area; MIF as a pro-inflammatory cytokine in the deep area. Their association with CRC was already suggested, as it was shown that a direct interaction between platelet and colon cancer cells promotes lung metastasis dissemination inducing EMT [41]. Moreover, MIF can modify the TME by promoting angiogenesis, inhibiting apoptosis, and stimulating fibroblast proliferation [60]. Lactotransferrin is also part of this class of inflammatory/immune-regulatory proteins. A down-regulation of lactotransferrin was found in CRC [39], contrary to our results, which suggested its tumor-suppressing action in CRC cells. Gal-3 is a ubiquitous protein with intra- and extra-cellular localizations, and it has also been found as a high-level circulating protein in CRC patients [77]. Moreover, during CRC progression its expression increases and it moves from the nucleus to the cytoplasm [77]. Gal-3 regulates several transduction pathways and exerts anti-apoptotic activity and pro- or anti-inflammatory effects depending on the tissue and subcellular localization, as indicated by GO annotations. The secreted Gal-3 interacts with the ECM and cell-surface glycans and it oligomerizes forming a lectin scaffold that supports the cell surface signaling receptors. This activity improves the survival of tumor cells under stress conditions, induces tumor cell detachment and migration, and modifies the cell composition of the TME [77]. It could be amazing to hypothesize that the lowest levels of Gal-3 determined in tumor tissues could be connected to its capacity for oligomerization when secreted in the TME. This hypothesis could explain the negative correlation between the Gal-3 level and the budding that we observed in the superficial tumor. Similarly to Gal-3, N-WASP and basigin may affect not only the ECM dynamics but also the inflammatory and immune conditions of the TME in CRC. In addition, CgA contributed to delineate the cluster of immune-regulatory proteins. Indeed, based on GO annotations, CgA can induce mast cell migration and degranulation. The usual processing of CgA generates several bioactive peptides, which regulate a broad spectrum of activities. However, the sequencing of tryptic peptides showed intact cleavage sites from which bioactive peptides are generated from the CgA pro-protein. Therefore, in our samples the pro-protein was intact. The lowest levels of CgA in both tumor regions might suggest a loss of activity and/or a down-expression of this protein in CRC, in accordance with a previous study [74]. IgGFc-binding protein and calcium-activated chloride channel regulator 1 (CaCC-1), identified as less-abundant proteins in both tumor samples, can be included in this cluster of proteins, even if they were not recognized by the enrichment analysis. The down-expression of IgGFc-binding protein, a tumor-suppressor capable of reducing cancer progression and metastatic behavior, as well as that of CaCC-1, has been evidenced in CRC [70,78]. Indeed, both proteins participate in protective immunity and inflammation. CaCC-1 is able to reduce CRC growth via the inhibition of Wnt/β-catenin signaling [70].

#### 4.1.3. Proteins Implicated in Metabolic Processes

**Figure 6:**
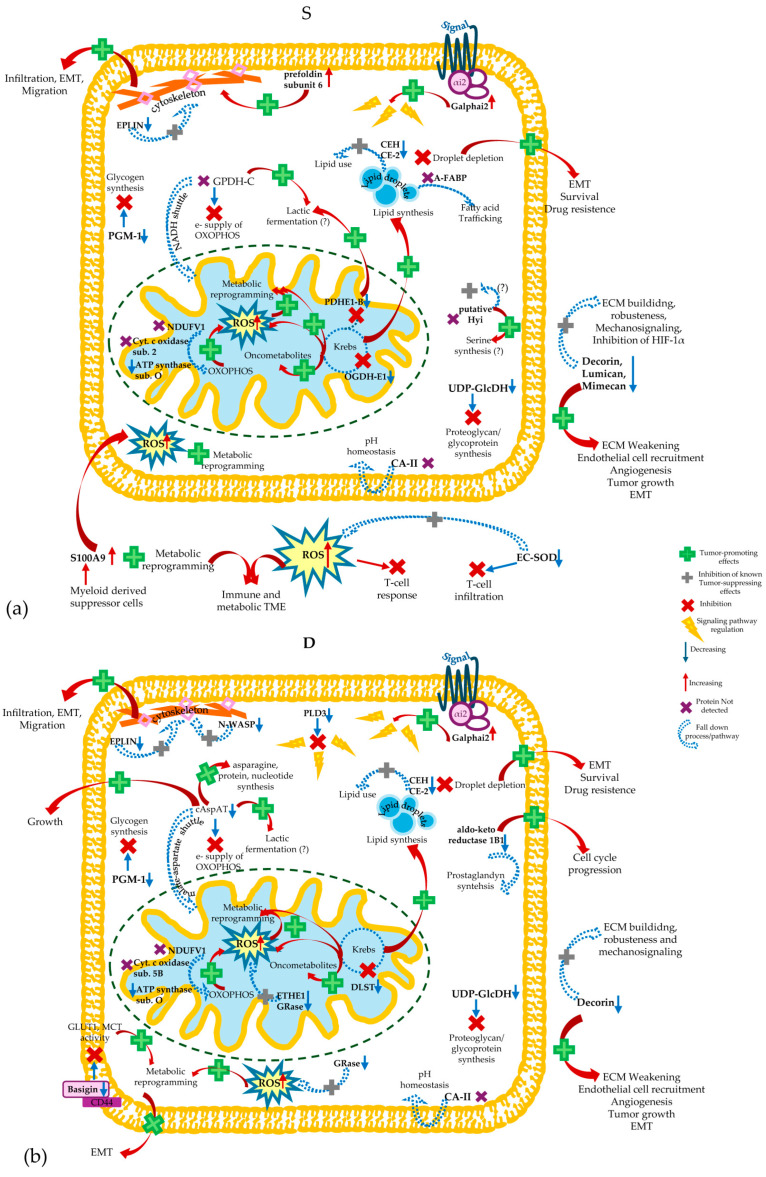
Representation of the metabolic implications, and their probable down- or up-regulation, relative to proteins significantly variated in S (**a**) or D (**b**) samples.

The majority of the analyzed proteins were involved directly or indirectly in metabolic processes. This was an expected result, because the metabolic reprogramming was closely connected to the initiation and progression of CRC. The activation of oncogenic pathways and the down-regulation of tumor suppressor genes reprogram several processes, such as glycolysis, glutaminolysis, one-carbon metabolism and lipid metabolism [4,5]. The anomalous metabolic reprogramming guarantees energy, nutrients, and redox conditions required to support the tumor growth and metastasis. The metabolic reprogramming of CRC cells is supplemented by dysbiosis of the gut microbiota and by impaired metabolism in TME cells. It was suggested that the alteration of the metabolite fluxes, signaling pathways, and regulatory processes may generate metabolic crosstalk among CRC cells, TME cells, and he intestinal microbiota, which favors CRC invasion and metastasis [4,5]. In this scenario, the components of the ECM in the tumor stroma can establish crosstalk with secreted proteins that regulate inflammation and innate immunity, as suggested by the interaction among basigin, Gal-3, and S100A9. The immunosuppressive microenvironment generated by the contribution of S100A9 supports the metabolic reprogramming of tumor cells and TME cells. Moreover, S100A9 released into the TME by monocytes and myeloid-derived suppressor cells can affect the carbohydrate and lipid metabolism and induce abnormal oxidative metabolism by the activation of NADPH oxidase of both tumor cells and their surrounding cells [93,94]. The production of reactive oxygen species (ROS) activates molecular pathways, promoting tumor development and abolishing the T-cell response to the tumor growth. Other proteins identified in this study may contribute to these events, which based on our results are prevalent in the tumor surface. EC-SOD was found to be a less-abundant protein in superficial tumor samples, in contrast to S100A9, leading to a lack of defense against ROS in the extracellular environment. Figure 6 represents the probable metabolic changes occurring on the surface of the tumor (panel a) and in the deep region (panel b), suggested by our results. It was demonstrated that the loss of extracellular EC-SOD promotes the TME, favoring cancer progression, while its overexpression inhibits tumor growth and metastasis [95]. Moreover, EC-SOD induces the vascular normalization and specific intra-tumor infiltration of effector T-cells by altering the WNT/β-catenin pathway in endothelial cells [95]. Even if it was found as a less-abundant protein in the tumor surface, EC-SOD showed a positive significant correlation with the worsening of the Dukes stage in this part of the tumor, suggesting that its tumor-suppressing effect is down-regulated in the low-stage tumor, while CRC cells could exploit EC-SOD for their ROS homeostasis with the stage progression, whereas in the deep part of the tumor, a down-regulation of mitochondrial antioxidant activities was revealed and associated with ETHE1 and glutathione reductase (GRase). They were both found to be less-abundant proteins in this tumor area (Figure 6, panel b), and they were both involved in glutathione metabolism. ETHE1 contributes to the containment of oxidative stress in mitochondria caused by hydrogen sulfide. Contrary to our results, it was shown that CRC cells suppress the toxic effects of hydrogen sulfide, overexpressing sulfide oxidation pathway enzymes and likely changing ETHE1’s cellular localization [48]. 

Our study, therefore, evidenced conditions that favor oxidative stress in CRC cells at the intracellular and extracellular levels. We could not establish if this event exerted positive or negative effects on CRC cells; however, we might suppose tumor-promoting effects from the increased conditions of oxidative stress. Indeed, it was reported that cancer cells can exploit the tumor-promoting effects of ROS with different mechanisms, and simultaneously implement repair systems against oxidative damage [96]. The principal source of altered ROS homeostasis in cells is the mitochondrial electron transport chain. In cancer cells, oncogenic pathways take over the electron transport chain enhancing its ROS production by increasing electron flow or by interrupting its organization [96]. This study showed that proteins implicated in OXOPHOS, as well as in the citric acid cycle, were overall less abundant in the tumor regions of CRC compared to healthy mucosa. NDUFV1, subunit 1 of the Complex I core of the electron transport chain, was not detected in any tumor sample, consistent with a recent study showing the down-regulated expression of Complex I in CRC [84]. Mutations and deletions in the mitochondrial DNA cause defective assembly and/or function of the complexes working in the electron transport chain [28]. Defective Complex I and cytochrome c oxidase are associated with CRC, contributing to ROS production and activating the related downstream signaling pathways [28]. Our investigation showed that both subunit 2 of cytochrome c oxidase, encoded by the mitochondrial genome, and subunit 5B, encoded by the nuclear genome, could be defective in CRC in different areas of the tumor (Figure 6). While defective subunit 2 was already associated with CRC [28], the result on subunit 5B was novel. In addition, we found ATP synthase subunit O with the lowest levels in the tumor, as evidenced in another proteomic study [68].

Other enzymes crucial for the mitochondrial bioenergetics were less abundant in tumor samples, including OGDH-E1 and DLST, components of the OGDH complex, and PDHE1-B, a component of the PDH complex. As observed for cytochrome c oxidase, different subunits of the OGDH complex showed significant quantitative changes in the different regions of the tumor: OGDH-E1 was less abundant on the surface and DLST in the deep tumor regions, both novel outcomes never evidenced before (Figure 6). This suggested the adoption of different strategies by the two different analyzed tissues of the CRC to reprogram the metabolite flux of the Krebs cycle. In the context of the mitochondrial redox homeostasis, the OGDH complex is a critical redox sensor. OGDH complex dysregulation contributes to increased ROS levels and interrupts substrate fluxes, leading to the production of oncometabolites, such as L-2-hydroxyglutarate, during the process of cancer pathogenesis and development [5,97] (Figure 6). Moreover, different factors during cancer growth, such as decreases in glucose-derived citrate, defective OGDH complexes, and isocitrate dehydrogenase, great glutamine demand, and the glutaminolysis process, support the reductive glutamine metabolism. Indeed, the accumulation of 2-oxoglutarate drives a reversion of the isocitrate dehydrogenase reaction in reductive carboxylation, which results in citrate accumulation followed by lipid synthesis [4,5,96] (Figure 6). Hypoxia amplifies these conditions and stabilizes HIF-1α, enhancing the accumulation of oncometabolites deriving from the Krebs cycle and impairing DNA methylation to remodel intracellular environments optimal for cancer growth. HIF-1α facilitates ubiquitination and proteolysis of the E1 subunit of the OGDH complex [5,97], an outcome in accordance with the OGDH-E1 variation determined in this study on the surface of the tumor.

Moreover, the levels of OGDH-E1 in the deep tumor tissue, even if similar to those in healthy tissue, increased in patients with a higher budding index. Like OGDH-E1, a defective DLST in the deep part of the tumor probably drives the same reversed Krebs cycle (Figure 6b). DLST was first associated with CRC in this study.

Furthermore, the defective PDH complex inhibits the mitochondrial pyruvate metabolism, and it was consistent with the defective OGDH complex in the superficial tumor regions (Figure 6). In addition, the failure to detect GPDH-C on the surface of the tumor was coherent with the down-regulation of PDH complex activity and suggested a re-oxidation of glycolytic NADH attributed to the lactic fermentation of cytosolic pyruvate (Figure 6) rather than through the electron transfer chain by the glycerophosphate shuttle. Our results were in contrast with the study of Krasnov et al., which associated increased levels of GPDH-C to CRC [30], but were coherent with a decreased mitochondrial carbohydrate metabolism and increased cytosolic use of glucose. Based on these results, glycolysis and the citric acid cycle appeared uncoupled in the CRC cells of the tumor surface, supporting the metabolic flux reprogramming occurring in CRC cells and favored the Warburg effect [5,97]. Despite this, the present study surprisingly did not highlight significant variations in glycolytic enzymes. L-lactate dehydrogenase chains A and B were detected in all the samples, as well as pyruvate kinase M, but it was not possible to distinguish between the M2 or M1 isoforms (Supplementary File S1). The only significant variation regarding the cytosolic metabolism of glucose was shown by PGM-1, identified as less abundant in the superficial tumor regions (Figure 6a). In addition, its Lys370-acetylated derivative was identified for the first time in this study. PGM-1 is essential to address glucose to glycogen synthesis; thus, a reduction in PGM-1 levels in CRC cells could stimulate cellular proliferation and tumor growth by enhancing the glycolytic pathway as previously proposed [34]. However, PGM-1 can play a tumor-promoting or tumor-suppressing role in an environment-dependent manner [34]. Regarding the lipid metabolism, the results suggested a down-regulation of some metabolic pathways in the two different regions of the tumor. Considering the GO annotations, the failure to detect A-FABP in superficial tumor samples indicated an impairment of the extracellular and/or intracellular utilization of fatty acids released by lipolysis from lipid droplets (Figure 6a). Consequentially, defective fatty acid trafficking may increase the accumulation of intracellular lipid droplets useful to support the survival, invasion, and drug resistance of CRC cells. From this point of view, our results were in accordance with the tumor-promoting action of A-FABP leading to EMT [4]. In our CRC samples, a stimulating effect on lipid droplet storage was also enabled by low levels of CEH and CE-2 in both the analyzed tumor regions (Figure 6). They are enzymes involved in the lipid and xenobiotic metabolism, as indicated by their GO annotations. Consistent with our results, they were found down-regulated in CRC and were indicated as tumor-suppressors in previous studies [72,73]. It was demonstrated that CEH inhibits CRC progression and invasion and promotes the apoptosis of CRC cells inducing fatty acid β-oxidation, the depletion of lipid droplets, and the consequent increased content of toxic ROS. The deep part of the tumor was also characterized by defective processes involving aldo-keto reductase 1B1 (Figure 6b), previously indicated as a tumor-promoter in CRC [44]. Aldo-keto reductase 1B1, involved in prostaglandin synthesis, could have a role in inflammation and the induction of cell cycle progression in CRC and its expression could change depending on the stages, types, and invasiveness of the tumor cells [44].

cAspAT was identified as a less-abundant protein in the deep tumor region, and this result can be rationalized by considering that in CRC cells of the internal tumor part, the defective transamination of cytosolic aspartate in oxalacetate may favor the use of aspartate in the synthesis of asparagine, proteins, and nucleotides promoting the cell growth (Figure 6b). As a consequence, the metabolic axis aspartate–oxalacetate–malate–pyruvate expected to contain the oxidative stress is disfavored [98], a condition consistent with our hypothesis that oxidative stress conditions may be implemented under the control of CRC cells. CRC cells can also utilize this axis to produce lactate [46]. It is remarkable that cAspAT, also known as AST1, participates in the malate–aspartate shuttle, which transfers electrons from glycolytic NADH to OHOPHOS. Therefore, we could suppose that defective cAspAT activity may contribute to the re-oxidation of cytosolic NADH by L-lactate-dehydrogenase (Figure 6b). Moreover, the results found on putative hydroxypyruvate isomerase (putative Hyi), which converts β-hydroxypyruvate in 2-hydroxy-3-oxopropanoate and participates in glyoxalate metabolism (GO annotation) were interesting. β-hydroxypyruvate, obtained by serine catabolism, can sustain gluconeogenesis, and through an inverse path can be precursor of the serine utilizing phosphoserine-AST1, an aminotransferase coupled to cAspAT activity [46]. Putative Hyi, not detected in any superficial tumor samples, was never associated with CRC before. Three hypotheses arise: (i) an alteration of the glyoxalate metabolism in CRC cells; (ii) an enhancement of the metabolic flux to serine synthesis necessary to obtain glycine, glutathione, NADPH, and nucleotides, suggesting probable tumor-promoting effects as consequences of the putative lost Hyi activity; (iii) a probable action of putative Hyi as a tumor-suppressor of CRC that is inhibited by cancerous cells specifically in the superficial region of the tumor (Figure 6a).

Regarding the proteoglycan metabolism, this study found significant decreased levels of UDP-GlcDH in tumor samples, as well as of lumican, decorin, and mimecan (Figure 6). With a not-defined mechanism, UDP-GlcDH might participate in CRC carcinogenesis as part of a functional network with several proteins involved in ECM regulation and with cancer-related functions, like A-FABP [80]. N-WASP, implicated in actin and cytoskeleton metabolism (Figure 6a), was found to be a less-abundant protein in the deep part of the tumor, and its abundance significantly decreased in the superficial tumor samples in relation to the worsening of the Dukes stage, consistent with a previous investigation [51]. Similarly to N-WASP, LIM domain-actin-binding protein 1 (also known as epithelial protein lost in neoplasm, EPLIN) is associated with the regulation of actin dynamics. We identified EPLIN as a less-abundant protein in both tumor regions, in accordance with previous studies evidencing that its loss affects CRC cell migration, invasion, and metastatic potential [59] (Figure 6). Prefoldin subunit 6 was another interesting protein involved in the cytoskeleton dynamics, especially in the correct folding of newly synthetized cytoskeletal proteins (GO annotations). It was significantly more abundant in superficial tumor samples (Figure 6b), a novel insight on the association between prefoldin family and CRC. Basigin was one of the most interesting proteins identified in this study, able to establish different protein interactions and to participate into several processes, including the regulation of metabolic processes/pathways. Basigin controls glucose and lactate membrane transport due to the association of the basigin–CD44 complex with GLUT1 and MCT, and it is required for their cell surface translocation and activity [91]. The low levels of basigin in the deep part of the tumor may affect the dependent switching of the Warburg effect in CRC cells, from classical to reverse, which depends on the transmembrane L-lactate distribution (Figure 6b). In addition, CA-II and PLD3 are proteins influencing metabolism regulation (Figure 6). In accordance with our results, CA-II was found to be down-regulated in CRC [71]. Carbonic anhydrase activity is needed by the tumor cells to maintain a neutral intracellular pH, while the pH of the TME decreases during tumor growth, interrupting physiological processes of the neighboring normal tissue. PLD3, which is involved in many signaling pathways (Figure 6b), was indicated as a CRC hallmark for the first time in this study, and it underlines the negative correlation exhibited by PLD3 with the budding index in the deep part of the tumor where the protein was shown to be a less-abundant protein.

Galphai2 belonging to the Galpha inhibitory subfamily of G proteins may evoke signaling through a broad range of effectors and regulate metabolic pathways (Figure 6). A recent study evidenced tumor-suppressing *GNAI2* mutations leading to β-catenin degradation and consequent decreased cell proliferation in CRC [82]. Probably, Galphai2 may show tumor-suppressing or promoting effects in a context-dependent manner. Our study did not identify Galphai2 variants, and the highest abundance demonstrated in both the two regions of the tumor suggested its tumor-promoting actions. Moreover, it is significant that this protein showed a positive correlation with the Dukes stage in the deep part of the tumor, suggesting that Galphai2 has prognostic potential.

#### 4.1.4. Other Interesting Proteins Potentially Hallmarks of CRC

##### Hallmarks Implicated in Nucleic Acid Processing

Several proteins identified in this study are involved in the homeostasis and metabolism of nucleic acids, DNA repair, posttranscriptional regulation of gene expression, mRNA processing, splicing and transport, and chromatin assembly, as indicated by their GO annotations for function and processes. They could be proposed as tumor-promoters since they were found to be more-abundant proteins either in the superficial region, such as WDR5, or in the deep area of the tumor, such as X-ray repair cross complementary 1 (XRCC1), NASP, heterogeneous nuclear ribonucleoprotein D-like (hnRNP D-like), histone H3.3, EF-2, and RNA-binding protein 34. XRCC1 and WDR5 were not present in healthy control tissues, suggesting their specific tumor-associated roles. *XRCC1* mutations occur in the precancerous stage of CRC and favor the progression from adenoma to carcinoma. It was suggested that this phenomenon is associated with the up-regulation of immune checkpoint expression that leads to T-cell dysfunction or exhaustion in the TME [65], so our finding on XRCC1 was consistent with the results obtained on S100A9 and EC-SOD. EF-2 is a crucial factor for the protein synthesis, which is typically dysregulated in CRC carcinogenesis. The EF-2 up-regulation in CRC and its importance for cancer progression and migration especially in stress conditions have been demonstrated [58]. NASP is a component of the multichaperone nucleosome-remodeling complex important for the assembly of chromatin after DNA replication. Due to the decreasing abundance in the deep tumor area in relation to the worsening of the Dukes stage, NASP could be proposed as novel CRC hallmark with tumor-promoting effects and useful for monitoring tumor extension.

##### Hallmarks Implicated in Protein Trafficking

Similarly to basigin, several proteins showing significant variations in the tumor samples regulate extra- and/or intracellular protein trafficking. Among them, sorting nexin-18 and GRASP-1 were the only proteins with significantly different levels between the surface and the deep part of the tumor. These results suggested a tumor-promoting action for GRASP-1 and tumor-suppressing action for sorting nexin-18 in the deep tumor region, where sorting nexin-18 showed the lowest abundance and GRASP-1 the highest. Consequently, in the same CRC cells the processes involving sorting nexin-18 and GRASP-1 could be down-regulated and up-regulated, respectively. Among those here identified, sorting nexin-18 was the only isoform showing significant variations in the tumor samples. It regulates membrane/intracellular protein trafficking and protein sorting in the endosomal system (GO annotations). Sorting nexin-18 was indicated as a marker discriminating primary CRC from the corresponding liver metastasis [24]. GRASP-1 is a guanine nucleotide exchange factor for the Ras family of small G proteins, and it forms a complex with glutamate receptor interacting protein 1 that modulates the glutamate receptor function. In addition, GRASP-1 could modulate the delivery and organization of transmembrane proteins at the membrane surfaces (GO annotations). A down-regulation of membrane trafficking and exocytosis was also evidenced by the results obtained on synaptogyrin-2, which was less abundant in the deep area of the tumor, contrary to a previous study reporting an up-regulation of this protein in CRC and evidencing its contribution to the immune infiltration in the TME [53]. Moreover, the synaptogyrin-2 abundance in the tumor surface showed a decreasing trend significantly correlated with the worsening of the Dukes stage and budding index. The results on these three proteins were novel in the CRC research field; they have not been associated so strongly and specifically to CRC before, and they inspire future targeted investigations.

Coatomer subunit beta (beta-COP) can mediate forward and reverse trafficking of proteins between the endoplasmic reticulum and the trans Golgi network. Beta-COP was found to be abundant in extracellular vesicles released by CRC cells cultured under hypoxia [55], and it has been identified as a hallmark capable of distinguishing two different populations of exosomes released from CRC cell-derived organoids [99]. From this perspective, the identification of beta-COP as a more-abundant protein in the deep part of the tumor could be associated with the ability of this CRC region to release exosomes/extracellular vesicles. These vesicles, transporting their protein/metabolite/lipid/mRNA content, might improve the TME ability to guarantee cancer survival, progression, and direct communications among the TME cells by connecting the cytoplasm of neighboring cells through membranous bridges [99].

##### Hallmarks Implicated in Regulation of Intra-/Extracellular Signaling Paths

Dual specificity protein phosphatase 3, also known as Vaccinia H1-related phosphatase (VHR), TAB182, four and a half LIM domains protein 1 (FHL-1), leucyl-cystinyl aminopeptidase, also known as oxytocinase (OTase), and mapK-regulated corepressor-interacting protein 1 (MCRIP1) were found as less-abundant proteins on the surface of the tumor. MCRIP1 was never detected in the S samples. Moreover, except for FHL-1, they were novel hallmarks potentially interesting in CRC. Epithelial-cell adhesion molecule (Ep-CAM) and 14-3-3 protein theta showed the lowest levels in both the tumor regions here analyzed. Most of these proteins often affect the growth and progression of tumors in a context-dependent manner, due to their capacity to participate in the regulation of several processes. Our results on Ep-CAM confirmed those of a previous study [76]. This protein, also known as CD326, gathers great interest in the recent CRC research as a potential biomarker for tumor-initiating cells and for identifying cancer stem cells within a tumor population, which is significant in CRC early diagnosis [100]. Ep-CAM is a membrane glycoprotein that can be cleaved, generating three proteoforms with different roles and localizations, namely cell membrane-sited, extra- and intracellular. The extracellular proteoform is implicated in cell–cell adhesion, while the intracellular form translocates into the nucleus where it interacts with several signaling proteins, including β-catenin. The detection of the Ep-CAM intracellular form in the nucleus correlates with increased tumor cell dissociation, EMT activation, and the potential for invasive CRC phenotypes [100]. The detection of both truncated forms of Ep-CAM correlates with advanced stages of CRC and vascular invasion. Finally, a complete loss of Ep-CAM in the membrane distinctly marks Lynch syndrome-associated CRC [100]. The present study identified the extracellular domain (high-MS/MS data deposited on ProteomeXchange repository). The role of Ep-CAM in tumor progression is still questionable, and the function of this marker could change in an environment-dependent manner. It would be interesting in a future study to investigate with targeted approaches based on HR-MS and immunochemical analyses the cellular localization and the ratio of the three possible proteoforms of Ep-CAM.

14-3-3 protein theta is a scaffold protein supporting the formation of multi-protein complexes and interactions with numerous phosphoproteins. Through this activity it can participate in the regulation of several cellular processes and signaling pathways (GO annotations). Our data showed for the first time that the isoform theta may be considered as a hallmark of CRC, due to the positive correlation found in the deep part of the tumor with the worsening of the Dukes stage.

ArgBP2 is an adapter protein for the assembling of signaling complexes in the cytoskeleton. ArgBP2 was associated with CRC for the first time in this study, where it was found to be more abundant in deep tumor samples. ArgBP2 may be an interesting protein marker to be investigated for its potentially tumor-promoting role and considering its significant positive correlation with the worsening of the Dukes stage on the surface of the tumor.

### 4.2. Identification of Highly Sensitive and Specific Classifying Proteins

RF classification analysis allowed us to build a machine-learning approach able to effectively discriminate the three types of tissues, superficial and deep tumor tissues and healthy mucosa, based on 21 selected proteins with the highest classification power. Indeed, RF classification analysis not only accurately classified samples into the three different groups but also identified which proteins, and to what degree, most effectively discriminated them. Unlike comparisons that use statistical tests, RF does not consider differences between means or distributions, but differences that discriminate individual samples [16,17]. The separation of the three groups was remarkably evident not only between the tumor regions and the healthy mucosa, but also between the deep and peripheral regions of the tumor from the same patient, despite the few differences found. The heatmap of the quantitative MS data evidenced differential patterns among the 91 proteins exhibiting significant variations in the comparative statistical test. Notably, the 21 proteins used for RF classification were distributed across all major clusters formed by the 91 proteins, suggesting that these 21 classification components covered the full range of relationships among the proteins varying among the three groups. The protein profiles of the non-tumor samples used as internal healthy controls showed greater homogeneity than the superficial and deep tumor regions, which were more varied among the individuals. This outcome is likely connected to the intrinsic heterogeneity of CRC cells, as well as differences in growth, aggressiveness, invasiveness, and staging, as demonstrated by the correlation analysis of proteomic data with some clinical characteristics of the patients. All these proteins exhibited significant variations by quantitative differential analysis, which provided suggestions for protein expression, regulation, or clearance. Furthermore, these proteins proved to be accurate, sensitive, and specific classifying factors for the different types of tissues analyzed, thus accurately distinguishing the superficial layer from the internal deep part of the tumor at the proteomic level. From this perspective, our approach provided novel insights and was shown to be effective in highlighting the intratumor heterogeneity typical of CRC [2,3]. Galphai2, CA-II, GRASP-1, sorting nexin-18, CEH, ADH1C, beta-COP, and IgGFc-binding protein were the most important proteins for classifying the three types of tissues analyzed.

The non-tumor tissue was well classified by Galphai2, CA-II, ADH1C, CEH, and IgGFc-binding protein, while ADH1B contributed with a lower MDA score. They are all involved in metabolic processes and regulation, except for IgGFc-binding protein. These proteins could be considered potential diagnostic markers useful during the diagnostic *iter* of CRC, as they can accurately classify healthy tissue even if taken from the same patient at a site distant from the tumor. Future studies should confirm these results by investigating the intestinal tissue from healthy individuals. Galphai2 was one of the most promising novel biomarkers of CRC; its highest concentration in cancer cells affects several biological processes downstream of the signaling pathways where it participates, and defective Galphai2 can provoke a dysregulation of metabolism in CRC cells. CA-II, ADH1C, CEH, IgGFc-binding protein, and ADH1B were all found to be less-abundant proteins in both regions of the tumor analyzed, suggesting their possible down-expression in CRC cells. Among them, IgGFc-binding protein was also a novel hallmark of suppression of the intestinal inflammatory/immune response that can be pursued in future studies.

GRASP-1 and MCRIP1 could be proposed as novel candidates in CRC biomarker discovery. They were able to classify tumor tissue with respect the non-tumor tissue, especially GRASP-1. The deep region of the tumor was accurately classified by sorting nexin-18 and beta-COP, while peptide YY (PYY), NID-1, and XRCC1 contributed weakly. Like sorting nexin-18, PYY was never detected in the deep region of the tumor and was identified as a less-abundant protein in superficial tumor tissue. PYY could not be associated with any cluster of proteins identifying biological processes/pathways; indeed, it is a gastrointestinal peptide inhibiting exocrine pancreatic secretion and intestinal mobility. From this study, PYY may potentially be considered a tumor-suppressor for CRC, as was suggested by a recent proteomic study demonstrating its low expression in CRC tissues and proposing it as a potential therapeutic target due to its ability to promote apoptosis and inhibit the proliferation, migration, and invasion of CRC-cell lines [79]. Beta-COP, NID-1, and XRCC1 could be considered tumor-promoters playing a role mainly in the part of the tumor that invades and progresses into the internal tissues of the colon, and from which CRC cells migrate, mature and metastasize. Since they are involved in biological processes regulating ECM organization, protein trafficking by extracellular vesicles, DNA repair, and events that inhibit apoptosis, modulate the TME, and support EMT and CRC cell detachment and migration, it was significant that these proteins classified the deep tumor tissue.

Superficial tumor regions were mainly categorized by OGDH-E1, proposed as a strong hallmark of the Krebs cycle diversion towards reductive glutamine metabolism and lipogenesis. We found two proteins, CaCC-1 and CgA, as discriminating factors of both healthy non-tumor and deep tumor tissues with respect to the tumor surface. Both proteins, showing the highest abundance in the healthy control samples, were good hallmarks of alteration in CRC cells’ regulatory processes implicated in inflammation and innate immunity. On the other hand, Ep-CAM, 14-3-3 protein theta, S100A9, and OTase categorized both healthy non-tumor and superficial tumor tissues.

## 5. Conclusions

The study, based on a high-throughput proteomic strategy and highly accurate statistical and enrichment analysis, not only confirmed potential biomarkers already proposed in other studies, but also revealed novel biomarkers able to distinguish the deep tumor region from the superficial one and from the healthy colon mucosa with high sensitivity and specificity. This study had some limitations, such as a small number of patients, the lack of technical verification of proteomic data (e.g., by Western blot for the most interesting proteins), and the absence of technical replication of the proteomic analysis. Despite these limitations, the results obtained appeared robust and reliable. Indeed, the application of rigorous analysis parameters, the high homogeneity of the protein profiles of the healthy control samples, the high accuracy of RF classification analysis in classifying each sample, the highly significant *p*-values obtained from statistical comparison (all < 0.01, mainly < 0.001), and the fold changes, confirmed that the experimental plan and the data analysis were conducted accurately and that the described data had scientific significance. The classifying proteins identified in this study can rightly be proposed as hallmarks of CRC cell heterogeneity. This finding is strengthened by the fact that the MW and KW tests confirmed the RF data, and, moreover, the data were obtained from the same subjects, increasing the reliability of the tests used. Our investigation revealed a huge and complex panel of protein hallmarks of CRC, most of which are implicated in catalytic and regulatory activities of metabolic pathways and processes that can be impaired as a consequence of the significant variations in specific key proteins. This outcome was especially evident regarding the mitochondrial dynamics and the redox homeostasis in the mitochondrial, cytosolic, and extracellular environment. Several of the protein hallmarks here identified might contribute to generate and modulate a metabolic and immune-suppressive TME that favors the cancer cell survival, progression, EMT, and metastatic potential of the tumor. In this context, the protein hallmarks implicated in the ECM assembly and modulation were revealed to be crucial, as well as those involved in cellular communication, protein trafficking, signaling pathways, and nucleic acid homeostasis. Moreover, the investigation highlighted different strategies adopted by cancer cells on the surface and in the deep zones, adapting metabolic, ECM, and immune checkpoints. This characteristic could be essential in developing effective therapeutics plans, and it suggests not only possible therapeutic targets but also the diagnostic potential of several proteins, especially those significantly correlated with classical clinic outcomes.

## Figures and Tables

**Table 1:** Demographic and clinical information, type of tumor, Dukes stage, and budding index of each patient included in the study.

Patients	Gender and Age	Dukes Stage	Budding
#4	F, 79 yr	B	3
#5	M, 78 yr	B	3
#6	F, 55 yr	B	2
#7	M, 83 yr	B	1
#8	M, 70 yr	B	2
#9	F, 81 yr	B	0
#11	M, 75 yr	B	1
#12	M, 66 yr	B	3
#13	F, 52 yr	C	1
#14	M, 52 yr	C	3
#15	M, 64 yr	C	3
#17	M, 76 yr	A	1
#19	M, 76 yr	C	1
#22	M, 80 yr	B	0
#23	F, 66 yr	B	2
#24	F, 80 yr	B	1

**Table 2:** Proteins (n = 91) with significant changes between groups (*p*-values < 0.05, cumulative FDR < 5%, FC ± 1.5). “*p*”: *p*-value; “change”: direction of the variation; “FC”: absolute fold change. Proteins are grouped based on the type and direction of the variation. When a short name of the protein is commonly used, it is reported. “ND”: failure to detect the protein. The 21 proteins selected by the Boruta method for the RF classification analysis are highlighted in gray. MW, Mann–Whitney test; KW, Kruskal–Wallis test. The GDA score from DisGeNET analysis is also indicated, as well as a previous reference (Ref.) associating the protein with CRC and the technique used for protein identification.

UniProt KB Code (Gene)	Protein	ND	MW S vs. D *p* Change FC			MW S vs. H *p* Change FC			MW D vs. H *p* Change FC			KW *p*	GDA Score	Ref. for CRC and Used Technique
**Proteins changing in the comparison S–D and D–H**														
Q4V328 (*GRIPAP1*)	GRASP-1	**ND in S**	0.00001	**S<D**	**>100**				0.0001	**D>H**	**>100**	0.00001		
Q96RF0 (*SNX18)*	Sorting nexin-18	**ND in D**	0.00001	**S>D**	**>100**				0.0003	**D<H**	**>100**	0.0001		LC–MS/MS [24]
**Proteins changing in the comparison S–H with S↓**														
P15090 (*FABP4)*	A-FABP	**ND in S**				0.0007	**S<H**	**>100**				0.0009	0.02	qPCR ^a^, IF ^b^ [4]
P02511 (*CRYAB*)	AlphaB-crystallin					0.0009	**S<H**	**>100**				0.0016	0.04	qPCR, IHC ^c^, WB ^d^ [25,26]
P50995 (*ANXA11*)	Annexin A11					0.0011	**S<H**	**2.8**					0.01	MALDI–TOF MS ^e^, IHC [27]
P00403 (*MT–CO2)*	Cytochrome c oxidase subunit 2	**ND in S**				0.0007	**S<H**	**>100**					0.5	Gene expression, WB, IHC [28]
P08294 (*SOD3*)	EC–SOD					0.0001	**S<H**	**4.1**						
Q13642 (*FHL1)*	FHL-1					0.0002	**S<H**	**10.1**				0.0003		mRNA expression [29]
P21695 (*GPD1)*	GPDH-C	**ND in S**				0.0001	**S<H**	**>100**				0.0004		MALDI–TOF [30]
P10915 (*HAPLN1)*	HPLPN1	**ND in S**				0.0003	**S<H**	**>100**				0.0004	0.5	Gene expression, qMSP ^f^ [31]
P51884 (*LUM*)	Lumican					0.0001	**S<H**	**7.2**				0.0004	0.01	WB, IHC, PCR [32]
P20774 (*OGN*)	Mimecan (osteoglycin)					0.0004	**S<H**	**11.7**				0.0004	0.03	IHC, WB, IF [33]
Q02218 (*OGDH)*	OGDH-E1					0.0008	**S<H**	**>100**				0.0002	0.01	LC–MS/MS, WB [5]
Q9UIQ6 (*LNPEP)*	OTase	**ND in S**				0.0000	**S<H**	**>100**				0.00001		
P11177 (*PDHB*)	PDHE1-B					0.0002	**S<H**	**>100**				0.0005		
P36871 (*PGM1*)	PGM-1					0.0009	**S<H**	**3.2**					0.3	qPCR, WB IHC [34]
Q5T013 (*HYI)*	Putative Hyi	**ND in S**				0.0007	**S<H**	**>100**						
Q9HD15 (*SRA1)*	SRA1	**ND in S**				0.0007	**S<H**	**>100**						WGCNA ^g^ [35]
Q9C0C2 (*TNKS1BP1*)	TAB182					0.0009	**S<H**	**1.6**						
P09936 (*UCHL1*)	UCH-L1					0.0002	**S<H**	**>100**				0.0004	0.04	WB, gene expression, qMSP [36]
P51452 (*DUSP3*)	VHR	**ND in S**				0.0007	**S<H**	**>100**						
**Proteins changing in the comparison S–H with S↑**														
P13674 (*P4HA1)*	4-PH alpha-1	**ND in H**				0.0003	**S>H**	**>100**				0.0006		LC–MS/MS [37]
P55957 (*BID*)	BH3-interacting domain death agonist					0.0012	**S>H**	**>100**					0.01	qPCR [38]
P02788 (*LTF*)	Lactotransferrin					0.0001	**S>H**	**6.0**				0.0004		qPCR, IHC, WB [39]
P49321 (*NASP*)	NASP					0.0012	**S>H**	**4.3**						
P48681 (*NES)*	Nestin	**ND in H**				0.0007	**S>H**	**>100**					0.01	IHC [40]
P02775 (*PPBP*)	PBP					0.0004	**S>H**	**2.2**					0.03	mRNA analysis, WB, IF [41]
O15212 (*PFDN6*)	Prefoldin subunit 6					0.0006	**S>H**	**2.8**						
Q9Y2Z0 (*SUGT1)*	Protein SGT1 homolog	**ND in H**				0.0003	**S>H**	**>100**				0.0008		
P05109 (*S100A8*)	S100-A8					0.0006	**S>H**	**7.7**						qPCR, WB, IHC [42]
P06702 (*S100A9*)	S100-A9					0.0000	**S>H**	**8.1**				0.0001	0.08	qPCR, WB, IHC [42]
P61964 (*WDR5)*	WDR5	**ND in H**				0.0003	**S>H**	**>100**				0.0008	0.01	qPCR, WB [43]
**Proteins changing in the comparison D–H with D↓**														
P15121 (*AKR1B1*)	Aldo-keto reductase 1B1								0.0008	**D<H**	**3.7**		0.05	gene expression, qMSP [44]
P17050 (*NAGA)*	Alpha-N-acetylgalactosaminidase								0.0009	**D<H**	**5.1**			
P35613 (*BSG*)	Basigin								0.0002	**D<H**	**4.8**	0.0011	0.06	WB [45]
P17174 *(GOT1*)	cAspAT								0.0002	**D<H**	**2.4**		0.01	qPCR [46]
Q9Y5K6 (*CD2AP*)	CD2-associated protein								0.0012	**D<H**	**3.7**		0.02	LC–MS/MS, IHC [47]
P10606 (*COX5B*)	Cytochrome c oxidase sub. 5B								0.0012	**D<H**	**4.5**			
Q13011 (*ECH1*)	DI (mitochondrial)								0.0007	**D<H**	**3.3**			
P36957 (*DLST*)	DLST								0.0008	**D<H**	**3.4**	0.0011		
O95571 (*ETHE1*)	ETHE1								0.0000	**D<H**	**4.0**	0.0002	0.02	IHC, WB, LC–MS/MS [48]
Q05315 (*CLC*)	Gal-10								0.0011	**D<H**	**4.9**		0.03	RNA analysis [49]
P00390 (*GSR*)	GRase								0.0002	**D<H**	**4.6**	0.0012	0.01	Enzymatic assay [50]
P06870 (*KLK1*)	Kallikrein-1								0.0006	**D<H**	**>100**	0.0003		
O00401 (*WASL*)	N-WASP								0.0009	**D<H**	**>100**		0.01	qPCR [51]
Q8IV08 (*PLD3)*	PLD3								0.0005	**D<H**	**>100**	0.0012		
O95716 (*RAB3D*)	Rab-3D								0.0010	**D<H**	**>100**			IHC, WB, qPCR [52]
O43760 (*SYNGR2)*	Synaptogyrin-2	**ND in D**							0.0007	**D<H**	**>100**	0.0015		GSEA ^h^ [53]
**Proteins changing in the comparison D–H with D↑**														
Q15121 (*PEA15*)	15 kDa phosphoprotein enriched in astrocytes								0.0009	**D>H**	**>100**	0.0011	0.02	WB, cell assays [54]
O94875 (*SORBS2*)	ArgBP2								0.0007	**D>H**	**>100**			
P53618 (*COPB1*)	Beta-COP								0.0002	**D>H**	**5.7**	0.0001	0.01	WB, IHC, LC–TOF MS [55]
P08572 (*COL4A2*)	Collagen alpha-2(IV) chain								0.0003	**D>H**	**>100**	0.0005	0.1	LC–MS/MS [37]
P02751 (*FN1*)	FN								0.0002	**D>H**	**2.9**	0.0004	0.4	RNA analysis, WB, IHC, GSEA [56]
P29279 (*CCN2)*	CTGF	**ND in H**							0.0003	**D>H**	**>100**	0.0012	0.08	WB, qPCR [57]
P13639 (*EEF2*)	EF-2								0.0002	**D>H**	**10.7**		0.01	MALDI–TOF, IHC, WB, ELISA [58]
P84243 (*H3–3A*)	Histone H3.3								0.0012	**D>H**	**>100**		0.01	RNA analysis [49]
O14979 (*HNRNPDL*)	hnRNP D-like								0.0001	**D>H**	**2.0**	0.0009	0.03	LC–MS/MS [59]
P14174 (*MIF*)	MIF								0.0007	**D>H**	**2.4**		0.03	IHC, WB, qPCR [60]
P14543 (*NID1*)	NID-1								0.0002	**D>H**	**>100**	0.000001	0.3	qPCR [61]
P42696 (*RBM34*)	RNA-binding protein 34								0.0009	**D>H**	**>100**	0.0013		qPCR, WB, IHC [62]
P50454 (*SERPINH1*)	Serpin H1								0.0004	**D>H**	**3.4**		0.01	DEGs ^i^, WGCNA [63]
Q8WUH6 (*TMEM263)*	Transmembrane protein 263	**ND in S/H**							0.0003	**D>H**	**>100**	0.000001	0.02	DEGs, ML ^j^ [64]
P18887 (*XRCC1)*	XRCC1	**ND in H**							0.0001	**D>H**	**>100**	0.00001	0.4	Gene expression, IHC [65]
**Proteins changing in the comparison D–H and S–H with D↓ and S↓**														
P27348 (*YWHAQ)*	14-3-3 protein theta					0.0001	**S<H**	**8.2**	0.0003	**D<H**	**8.3**	0.0001		
P00325 (*ADH1B*)	ADH1B					0.0010	**S<H**	**9.5**	0.0013	**D<H**	**8.3**	0.0006	0.4	Gene expression [66]
P00326 (*ADH1C)*	ADH1C					0.00001	**S<H**	**22.3**	0.00001	**D<H**	**26.3**	0.00001	0.06	DEGs, ML, IHC, WB, qPCR [67]
P48047 (*ATP5PO*)	ATP synthase subunit O					0.0008	**S<H**	**16.5**	0.0013	**D<H**	**3.5**	0.0004		LC–MS/MS, qPCR, WB [68]
O75363 (*BCAS1*)	Breast carcinoma-amplified sequence 1					0.0011	**S<H**	**3.5**	0.00001	**D<H**	**7.4**	0.0001		qPCR [69]
A8H7I4 (*CLCA1*)	CaCC-1					0.00001	**S<H**	**9.2**	0.00001	**D<H**	**22.1**	0.00001	0.06	ELISA, IHC, WB [70]
P00918 (*CA2*)	CA-II					0.00001	**S<H**	**5.7**	0.00001	**D<H**	**8.0**	0.00001	0.02	IHC [71]
O00748 (*SULT2*)	CE-2					0.0006	**S<H**	**9.2**	0.0004	**D<H**	**10.9**	0.0004	0.03	WB [72]
P34913 *(EPHX2*)	CEH					0.0002	**S<H**	**4.8**	0.00001	**D<H**	**>100**	0.00001		IHC, qPCR, WB [73]
P10645 (*CHGA*)	CgA, pro-protein					0.0001	**S<H**	**8.7**	0.00001	**D<H**	**18.2**	0.00001	0.03	ML of gene data, qPCR [74]
P07585 (*DCN*)	Decorin					0.0002	**S<H**	**9.7**	0.0008	**D<H**	**5.7**	0.0001	0.08	IF [75]
P16422 (*EPCAM*)	Ep-CAM					0.0001	**S<H**	**>100**	0.0001	**D<H**	**>100**	0.00001	0.1	IHC [76]
Q9UHB6 (*LIMA1*)	EPLIN					0.0010	**S<H**	**4.0**	0.0000	**D<H**	**5.6**	0.0001	0.1	LC–MS/MS [59]
P17931 (*LGALS3*)	Gal-3					0.0008	**S<H**	**2.2**	0.0007	**D<H**	**2.5**	0.0008	0.1	IHC [77]
Q9Y6R7 (*FCGBP*)	IgGFc-binding protein					0.00001	**S<H**	**9.8**	0.00001	**D<H**	**35.7**	0.00001		DEGs and ML [78]
P10082 (*PYY)*	PYY	**ND in D**				0.0010	**S<H**	**>100**	0.0000	**D<H**	**>100**	0.000001	0.01	LC–MS/MS, qPCR, IHC [79]
Q15661 (*TPSAB1*)	Tryptase-1					0.0003	**S<H**	**10.1**	0.0002	**D<H**	**6.5**	0.0003		
O60701 (*UGDH*)	UDP-GlcDH					0.0001	**S<H**	**3.4**	0.0000	**D<H**	**5.0**	0.0001		qPCR, WB [80]
**Proteins changing in the comparison D–H and S–H with D↑ and S↑**														
Q15582 (*TGFBI*)	Beta ig-h3					0.0008	**S>H**	**3.1**	0.0000	**D>H**	**2.7**	0.0001	0.02	LC–MSMS, GSEA [81]
P04899 (*GNAI2*)	Galphai2					0.00001	**S>H**	**6.3**	0.0003	**D>H**	**5.4**	0.00001		IF, WB, qPCR [82]
**Proteins changing in KW comparisons among all three groups but not in MW pairwise comparisons**														
Q9NZK5 (*ADA2)*	Adenosine deaminase 2	**N.D in D**										0.0006		
P35611 (*ADD1*)	Alpha-adducin											0.0012	0.01	Gene expression [83]
P0C0L5 (*C4B)*	Complement C4-B	**ND in S/H**										0.0013		
Q96S86 (*HAPLN3)*	HPLPN3	**ND in D**										0.0009		
C9JLW8 (*MCRIP1)*	MCRIP1	**ND in S**										0.0001		
P49821 (*NDUFV1)*	NDUFV1	**ND in S/D**										0.0003		WB, enzyme assay [84]
Q96IQ7 (*VSIG2*)	VSIG2											0.0015		WGCNA [85]
O75312 (ZPR1)	Zinc finger protein ZPR1 (ZPR1)	**ND in S**										0.0005		

^a^ qPCR, quantitative real-time polymerase chain reaction; ^b^ IF, immunofluorescence; ^c^ IHC, immunohistochemistry; ^d^ WB, Western blot; ^e^ MALDI–TOF MS, matrix-assisted laser desorption ionization time of flight MS; ^f^ qMSP, quantitative methylation analysis; ^g^ WGCNA, gene co-expression network analysis; ^h^ GSEA, gene set enrichment analysis; ^i^ DEGs, differentially expressed genes; ^j^ ML, machine-learning analysis.

**Table 3:** Networks of the biological processes (GO) or pathways (Reactome) with FDR < 0.05 obtained by including all the 91 proteins listed in Table 2. GO and/or Reactome entries, FDR, and implicated proteins are reported. When commonly used, short names are indicated.

Biological Processes/ Pathways	GO/Reactome Entry (FDR)	Proteins
Degradation of the ECM	**REAC:R-HSA-3560782 ** (0.02)	Decorin; lumican; mimecan; basigin; collagen alpha-2(IV) chain; FN; NID-1, tryptase-1
ECM proteoglycans	**REAC:R-HSA-3000178** (0.01)	Decorin; lumican; HPLPN1; collagen alpha-2(IV) chain; FN
ECM organization	**REAC:R-HSA-1474244** (0.003)	Decorin; lumican; HPLPN1; basigin; collagen alpha-2(IV) chain; FN; tryptase-1; 4-PH alpha-1; NID-1; serpin H1
Aerobic respiration/ Cellular respiration	**GO:0009060/GO:0045333** (0.02)	PDHE1-B; OGDH-E1; ATP synthase subunit O; cytochrome c oxidase subunit 2; cytochrome c oxidase sub. 5B; DLST; NDUFV1
Generation of precursor metabolites and energy	**GO:0006091** (0.02)	PDHE1-B; OGDH-E1; DLST; ATP synthase subunit O; cytochrome c oxidase subunit 2; cytochrome c oxidase sub. 5B; NDUFV1; PGM-1; ADH1C; ADH1B
Citric acid cycle and respiratory electron transport	**REAC:R-HSA-1428517** (0.003)	PDHE1-B; OGDH-E1; DLST; ATP synthase subunit O; cytochrome c oxidase subunit 2; cytochrome c oxidase sub. 5B; NDUFV1; basigin
2-oxoglutarate metabolic process	**GO:0006103** (0.02)	OGDH-E1; DLST; cAspAT
Histone succinylation/ Protein succinylation	**GO:0106077/GO:0018335** (0.03)/(0.04)	OGDH-E1; DLST
Metabolism	**REAC:R-HSA-1430728** (0.01)	PDHE1-B; OGDH-E1; DLST; cAspAT; ATP synthase subunit O; cytochrome c oxidase subunit 2; cytochrome c oxidase sub. 5B; NDUFV1; PGM-1; GPDH-C; ETHE1; ADH1C; ADH1B; CEH; CE-2; A-FABP; aldo-keto reductase 1B1; UDP-GlcDH; decorin; lumican; mimecan; CA-II; basigin; Galphai2; PLD3; N-WASP
Neutrophil chemotaxis	**GO:0030593** (0.03)	S100A8; S100A9; Gal-3; PBP; basigin
Leukocyte chemotaxis/ Myeloid leukocyte migration	**GO:0030595/GO:0097529** (0.02)	S100A8; S100A9; Gal-3; PBP; basigin; CgA; MIF
Leuckocyte migration	**GO:0050900** (0.04)	S100A8; S100A9; Gal-3; PBP; basigin; CgA; MIF; N-WASP
Neutrophil aggregation	**GO:0070488** (0.02)	S100A8; S100A9
Metal sequestration by antimicrobial proteins	**REAC:R-HSA-6799990** (0.002)	Lactotransferrin; S100A8; S100A9
Sequestering of zinc ion	**GO:0032119** (0.02)	S100A8; S100A9

**Table 4:** Key nodes of the backbone network that showed varied levels from statistical analysis. Pure bottleneck proteins are evidenced by an asterisk.

Gene	Closeness Centrality	Degree	Betweenness Centrality	Protein
*COPB2* *	0.36	6	0.002	Beta-COP
*DPP4* *	0.37	5	0.0001	ADABP
*EEF2*	0.52	44	0.022	EF-2
*FN1*	0.51	37	0.025	FN
*GOT1* *	0.32	5	0.0002	cAspAT
*IGHV3–43D* *	0.31	2	0.0001	Immunoglobulin heavy variable 3–43D
*LGALS3* *	0.43	12	0.004	Gal-3
*LTF* *	0.38	10	0.003	Lactotransferrin
*PDHB* *	0.41	9	0.0003	PDHE1-B
*WASL* *	0.41	14	0.003	N-WASP
*WDR5* *	0.41	9	0.002	WD repeat-containing protein 5

## Data Availability

The mass spectrometry proteomics data have been deposited into the ProteomeXchange Consortium via the PRIDE [1] partner repository with the dataset identifier PXD050863.

## Supplementary Materials

The following supporting information can be downloaded at https://www.mdpi.com/article/10.3390/cells13161311/s1. Supplementary File S1: Proteomic data concerning the 2009 proteins identified and quantified; Supplementary File S2: Table S1: Nineteen proteins with significant variation in the comparison among the three groups listed with the same order used for Table 2. Uniprot-KB codes and genes are indicated. The main GO ID annotations for the molecular function and biological processes reported were obtained from UniprotKB human data-bank provided by QuickGO available in the EMBL-EBI website (https://www.ebi.ac.uk/QuickGO/annotations); Table S2: Mean decrease accuracy scores of the three groups (S, D, and H). The relatively higher MDA scores for each protein are highlighted; Table S3: Results of the correlation analysis between the Dukes stage, budding index, and the LFQ abundances of the proteins (n = 91) with significant changes between groups. Spearman (Sp) Pearson (Pe) tests were used depending on whether the data were normally distributed or not; *p*-values and r coefficient are reported for each type of tissue (S, D, and H). In bold are the proteins that contribute to the RF classification; Figure S1: Topology of the backbone network. It consisted of 196 nodes with high betweenness centrality values and 1787 edges. The size of the nodes corresponds to their betweenness centrality values. Pink colored nodes are proteins with levels varied from the statistical analysis; Supplementary File S3: GO/Reactome entries and FDR from the functional analysis of all the 2009 proteins identified.

## Abbreviations

4-PH alpha-1 = prolyl 4-hydroxylase subunit alpha-1; *ACTB* = actin; ADH1B = alcohol dehydrogenase 1B; ADH1C = alcohol dehydrogenase 1C; ArgBP2 = Arg-binding protein 2, also known as sorbin-SH3 domain-containing protein; beta ig-h3 = transforming growth factor-beta-induced protein ig-h3; Beta-COP = coatomer subunit beta; CA-II = carbonic anhydrase 2; CaCC-1 = Calcium-activated chloride channel regulator 1; CAFs = cancer-associated fibroblasts; cAspAT = Cytoplasmic aspartate aminotransferase; CD44 = Cluster of Differentiation 44; CEH = bifunctional/cytosolic epoxide hydrolase 2; CgA = chromogranin-A; CRC = colorectal cancer; CTGF = connective tissue growth factor; DI = Δ3,5-Δ2,4-dienoyl-CoA isomerase; DLST = Dihydrolipoyllysine-residue succinyltransferase; ECM = extracellular matrix; EC-SOD = extracellular superoxide dismutase; EGFR = endothelial growth factor receptor; EF-2 = elongation factor 2; EMT = epithelial–mesenchymal transition; Ep-CAM = epithelial cell adhesion molecule; EPLIN = epithelial protein lost in neoplasm, also known as LIM domain-actin-binding protein 1; FHL-1 = four and a half LIM domains protein 1; FN = fibronectin; Gal-3 = Galectin-3; Galphai2 = guanine nucleotide binding protein G(i) subunit alpha 2; GAPDH = Glyceraldehyde-3-phosphate dehydrogenase; GDA = Gene–Disease Association; GO = gene ontology; GPDH-C = cytoplasmic glycerol-3-phosphate dehydrogenase; GRase = glutathione reductase; GRASP-1 = glutamate receptor interacting protein 1-associated protein 1; HIF-1α = hypoxia inducible factor-1α; hnRNP D-like = heterogeneous nuclear ribonucleoprotein D-like; HPLPN1 = hyaluronan proteoglycan link protein 1; HR = high-resolution; HSP90AA1 = Heat shock protein 90-alpha; IgGFc-binding protein = protein binding the Fc portion of IgG; KW = Kruskal–Wallis test; LFQ = label-free quantification; MCRIP1 = mapk-regulated corepressor-interacting protein 1; MDA = mean decrease accuracy; MIF = migration inhibitory factor; MW = Mann–Whitney test; NASP = nuclear autoantigenic sperm protein; NDUFV1 = NADH dehydrogenase ubiquinone flavoprotein subunit 1; NID-1 = nidogen-1; N-WASP = neural Wiskott–Aldrich syndrome protein; OGDH-E1 = 2-oxoglutarate dehydrogenase subunit E1; OTase = oxytocinase, also known as leucyl-cystinyl aminopeptidase; OXOPHOS = oxidative phosphorylation; PBP = platelet basic protein PBP; PDHE1-B = Pyruvate dehydrogenase E1 sub. beta; PGM-1 = Phosphoglucomutase-1; PLD3 = 5′–3′ exonuclease phospholipase D3; PPI = protein–protein interactions; PTMs = post-translational modifications; Putative Hyi = putative hydroxypyruvate isomerase; PYY = peptide YY; RF = Random Forest; ROS = reactive oxygen species; SR-alpha = signal recognition particle receptor subunit alpha; TAB182 = 182 kDa tankyrase-1-binding protein; TME = tumor microenvironment; UDP-GlcDH = UDP-glucose 6-dehydrogenase; VEGF = vascular endothelial growth factor; VHR = Vaccinia H1-related phosphatase, also known as dual specificity protein phosphatase 3; VSIG2 = V-set-immunoglobulin domain-containing protein 2; WDR5 = WD repeat-containing protein 5; XRCC1 = X-ray repair cross complementary 1.
