# Supplementary material for: Combined High—Throughput Proteomics and Random Forest Machine-Learning Approach Differentiates and Classifies Metabolic, Immune, Signaling and ECM Intra-Tumor Heterogeneity of Colorectal Cancer
Source: Cells. 2024 Aug 6;13(16):1311. doi: 10.3390/cells13161311 (PMC11352245; doi:10.3390/cells13161311)

Table S1. Nineteen proteins with significant variation in the comparison among the three groups listed with the same order used for Table 2. Uniprot-KB codes and genes are indicated. The main GO ID annotations for the molecular function and biological processes reported were obtained from UniprotKB human data-bank provided by QuickGO available in the EMBL-EBI website (https://www.ebi.ac.uk/QuickGO/annotations).

Protein name	Uniprot KB	*Gene*	GO ID Annotations for molecular function	GO ID Annotations for biological process
GRASP-1	Q4V328	*GRIPAP1*	GO:0005515 - protein binding<br>GO:0005085 - guanyl-nucleotide exchange factor activity	GO:0098887 - neurotransmitter receptor transport, endosome to postsynaptic membrane
Sorting nexin-18	Q96RF0	*SNX18*	GO:0005515 - protein binding<br>GO:0035091 - phosphatidylinositol binding	GO:0006897 – endocytosis<br>GO:0015031 – protein transport
A-FABP	P15090	*FABP4*	GO:0005324 - long-chain fatty acid transmembrane transporter activity	GO:0015909 - long-chain fatty acid transport
AlphaB-crystallin	P02511	*CRYAB*	GO:0005515 - protein binding<br>GO:0006457 – protein folding	GO:0043066 - negative regulation of apoptotic process
Annexin A11	P50995	*ANXA11*	GO:0005515 - protein binding<br>GO:0005544 - calcium-dependent phospholipid binding	GO:0006909 - phagocytosis
Cytochrome c oxidase subunit 2	P00403	*MT-CO2*	GO:0005515 - protein binding<br>GO:0004129 - cytochrome-c oxidase activity	GO:0006123 - mitochondrial electron transport, cytochrome c to oxygen
EC-SOD	P08294	*SOD3*	GO:0005515 - protein binding<br>GO:0004784 - superoxide dismutase activity	GO:0001666 - response to hypoxia<br>GO:0006979 - response to oxidative stress
FHL-1	Q13642	*FHL1*	GO:0005515 - protein binding<br>GO:0044325 - transmembrane transporter binding	GO:0007517 - muscle organ development<br>GO:0003254 - regulation of membrane depolarization<br>GO:0030154 – cell differentiation<br>GO:0030308 - negative regulation of cell growth
GPDH-C	P21695	*GPD1*	GO:0141152 - glycerol-3-phosphate dehydrogenase (NAD+) activity	GO:0006072 - glycerol-3-phosphate metabolic process<br>GO:0006127 - glycerophosphate shuttle
HPLPN1	P10915	*HAPLN1*	GO:0030021 - extracellular matrix structural constituent conferring compression resistance	GO:0001501 - skeletal system development<br>GO:0007155 - cell adhesion
Lumican	P51884	*LUM*	GO:0005515 - protein binding<br>GO:0030021 - extracellular matrix structural constituent conferring compression resistance	GO:0030199 - collagen fibril organization
Mimecan (osteoglycin)	P20774	*OGN*	GO:0030021 - extracellular matrix structural constituent conferring compression resistance<br>GO:0005515 - protein binding	GO:0007165 - signal transduction
OGDH-E1	Q02218	*OGDH*	GO:0004591 - oxoglutarate dehydrogenase (succinyl-transferring) activity	GO:0006103 - 2-oxoglutarate metabolic process
OTase	Q9UIQ6	*LNPEP*	GO:0005515 - protein binding	GO:0006508 - proteolysis

Protein name	Uniprot KB	*Gene*	GO ID Annotations for molecular function	GO ID Annotations for biological process
PDHE1-B	P11177	*PDHB*	GO:0004177 - aminopeptidase activity<br>GO:0004739 - pyruvate dehydrogenase (acetyl-transferring) activity<br>GO:0005515 - protein binding	GO:0006086 - acetyl-CoA biosynthetic process from pyruvate
PGM-1	P36871	*PGM1*	GO:0004614 - phosphoglucomutase activity	GO:0006006 - glucose metabolic process
Putative Hyi	Q5T013	*HYI*	GO:0005515 - protein binding<br>GO:0008903 - hydroxypyruvate isomerase activity	GO:0046487 - glyoxylate metabolic process
SRA1	Q9HD15	*SRA1*	GO:0002153 - steroid receptor RNA activator RNA binding<br>GO:0030374 - nuclear receptor coactivator activity	GO:0045893 - positive regulation of DNA-templated transcription<br>GO:0007346 - regulation of mitotic cell cycle<br>GO:0030154 - cell differentiation<br>GO:0042981- regulation of apoptotic process
TAB182	Q9C0C2	*TNKS1BP1*	GO:0005515 - protein binding GO:0071532 – ankyrin repeat binding	GO:0006302 - double-strand break repair
UCH-L1	P09936	*UCHL1*	GO:0005515 - protein binding<br>GO:0004843 - cysteine-type deubiquitinase activity	GO:0006511 - ubiquitin-dependent protein catabolic process
VHR	P51452	*DUSP3*	GO:0004725 - protein tyrosine phosphatase activity<br>GO:0033549 - MAP kinase phosphatase activity	GO:0035335 - negative regulation of JNK cascade<br>GO:0050860 - negative regulation of T cell receptor signaling pathway<br>GO:0070373 - negative regulation of ERK1 and ERK2 cascade
4-PH alpha-1	P13674	*P4HA1*	GO:0004656 - procollagen-proline 4-dioxygenase activity<br>GO:0031418 - L-ascorbic acid binding	GO:0030199 - collagen fibril organization
BH3-interacting domain death agonist (BID)	P55957	*BID*	GO:0005515 - protein binding	GO:0090200 - positive regulation of release of cytochrome c from mitochondria<br>GO:0043065 - positive regulation of apoptotic process<br>GO:0031334 - positive regulation of protein-containing complex assembly
Lactotransferrin	P02788	*LTF*	GO:0005515 - protein binding<br>GO:0005506 - iron ion binding	GO:0031640 - killing of cells of another organism<br>GO:0001817 - regulation of cytokine production
NASP	P49321	*NASP*	GO:0005515 - protein binding<br>GO:0042393 - histone binding	GO:0006335 - DNA replication-dependent chromatin assembly<br>GO:0006334 - nucleosome assembly<br>GO:0015031 - protein transport
Nestin	P48681	*NES*	GO:0005515 - protein binding<br>GO:0019215 - intermediate filament binding	GO:0007399 - nervous system development<br>GO:0030844 - positive regulation of intermediate filament depolymerization<br>GO:0032091 - negative regulation of protein binding<br>GO:0072089 - stem cell proliferation
PBP	P02775	*PPBP*	GO:0005515 - protein binding<br>GO:0008009 - chemokine activity<br>GO:0005125 - cytokine activity	GO:0006935 – chemotaxis<br>GO:0006952 - defense response<br>GO:0006954 - inflammatory response

Protein name	Uniprot KB	*Gene*	GO ID Annotations for molecular function	GO ID Annotations for biological process
			GO:0005355 - glucose transmembrane transporter activity<br>GO:0045236 - CXCR chemokine receptor binding	GO:0006955 - immune response<br>GO:0051781 - positive regulation of cell division
Prefoldin subunit 6	O15212	*PFDN6*	GO:0005515 - protein binding<br>GO:0051082 - unfolded protein binding	GO:0006457 - protein folding<br>GO:0050821 - protein stabilization
S100A8	P05109	*S100A8*	GO:0005515 - protein binding GO:0005509 - calcium ion binding	GO:0002523 - leukocyte migration involved in inflammatory response<br>GO:0006954 - inflammatory response<br>GO:0030593 - neutrophil chemotaxis<br>GO:0032496 - response to lipopolysaccharide<br>GO:0070488 - neutrophil aggregation
S100A9	P06702	*S100A9*	GO:0005515 - protein binding GO:0005509 - calcium ion binding	GO:0002523 - leukocyte migration involved in inflammatory response<br>GO:0006954 - inflammatory response<br>GO:0030593 - neutrophil chemotaxis<br>GO:0034121 - regulation of toll-like receptor signaling pathway<br>GO:0061844 - antimicrobial humoral immune response mediated by antimicrobial peptide<br>GO:0070488 - neutrophil aggregation
WDR5	P61964	*WDR5*	GO:0005515 - protein binding<br>GO:0035064 - methylated histone binding	GO:0051302 -regulation of cell division<br>GO:0051726 - regulation of cell cycle<br>GO:0006355 - regulation of DNA-templated transcription
aldo-keto reductase 1B1	P15121	*AKR1B1*	GO:0004032 - aldose reductase (NADPH) activity<br>GO:0016491 - oxidoreductase activity<br>GO:0047655 - allyl-alcohol dehydrogenase activity<br>GO:0052650 - all-trans-retinol dehydrogenase (NADP+) activity	GO:0046370 - fructose biosynthetic process<br>GO:0002070 - epithelial cell maturation<br>GO:0005975 - carbohydrate metabolic process<br>GO:0006629 - lipid metabolic process<br>GO:0006693 - prostaglandin metabolic process<br>GO:0006700 - C21-steroid hormone biosynthetic process
Alpha-N-acetylgalactosaminidase (alpha-NAGA)	Q9Y2Z0	*SUGT1*	GO:0005515 - protein binding<br>GO:0051087 - protein-folding chaperone binding	GO:0051382 - kinetochore assembly<br>GO:0007051 - spindle organization<br>GO:0014841 - skeletal muscle satellite cell proliferation<br>GO:0031647 - regulation of protein stability
Basigin	P35613	*BSG*	GO:0005515 - protein binding<br>GO:0001618 - virus receptor activity<br>GO:0010575 - positive regulation of vascular endothelial growth factor production	GO:0072659 - protein localization to plasma membrane<br>GO:1904466 - positive regulation of matrix metallopeptidase secretion<br>GO:0001525 – angiogenesis<br>GO:0007166 - cell surface receptor signaling pathway

Protein name	Uniprot KB	*Gene*	GO ID Annotations for molecular function	GO ID Annotations for biological process
cAspAT	P17174	*GOT1*	GO:0004069 - L-aspartate:2-oxoglutarate aminotransferase activity<br>GO:0004609 - phosphatidylserine decarboxylase activity	GO:0006114 - glycerol biosynthetic process<br>GO:0006532 - aspartate biosynthetic process<br>GO:0006094 - gluconeogenesis<br>GO:0006103 - 2-oxoglutarate metabolic process<br>GO:0006107 - oxaloacetate metabolic process<br>GO:0006520 - amino acid metabolic process<br>GO:0009058 - biosynthetic process
CD2-associated protein	Q9Y5K6	*CD2AP*	GO:0005515 - protein binding<br>GO:0017124 - SH3 domain binding<br>GO:0003779 - actin binding	GO:0007015 - actin filament organization<br>GO:0001771 - immunological synapse formation
Cytochrome c oxidase sub.5B	P10606	*COX5B*	GO:0005515 - protein binding<br>GO:0004129 - cytochrome-c oxidase activity	GO:0006123 - mitochondrial electron transport, cytochrome c to oxygen<br>GO:0006119 - oxidative phosphorylation
DI	Q13011	*ECH1*	GO:0005515 - protein binding<br>GO:0016853 - isomerase activity	GO:0006631 - fatty acid metabolic process
DLST	P36957	*DLST*	GO:0005515 - protein binding<br>GO:0004149 - dihydrolipoyllysine-residue succinyltransferase activity	GO:0006099 - tricarboxylic acid cycle<br>GO:0006091 - generation of precursor metabolites and energy<br>GO:0006103 - 2-oxoglutarate metabolic process<br>GO:0006104 - succinyl-CoA metabolic process
ETHE1	O95571	*ETHE1*	GO:0050313 - sulfur dioxygenase activity<br>GO:0005515 - protein binding	GO:0006749 - glutathione metabolic process<br>GO:0070813 - hydrogen sulfide metabolic process
Gal-10	Q05315	*CLC*	GO:0030246 - carbohydrate binding<br>GO:0004622 - lysophospholipase activity	GO:0002667 - regulation of T cell anergy<br>GO:0002724 - regulation of T cell cytokine production<br>GO:0070231 - T cell apoptotic process
Grase	P00390	*GSR*	GO:0004362 - glutathione-disulfide reductase (NADPH) activity	GO:0006749 - glutathione metabolic process<br>GO:0045454 - cell redox homeostasis
Kallikrein-1	P06870	*KLK1*	GO:0004252 - serine-type endopeptidase activity	GO:0006508 - proteolysis<br>GO:0003073 - regulation of systemic arterial blood pressure
N-WASP	O00401	*WASL*	GO:0005515 - protein binding<br>GO:0003779 - actin binding	GO:0030036 - actin cytoskeleton organization<br>GO:0006900 - vesicle budding from membrane
PLD3	Q8IV08	*PLD3*	GO:0005515 - protein binding<br>GO:0045145 - single-stranded DNA 5'-3' DNA exonuclease activity<br>GO:0004630 - phospholipase D activity	GO:1900015 - regulation of cytokine production involved in inflammatory response<br>GO:0002376 - immune system process<br>GO:0006259 - DNA metabolic process

Protein name	Uniprot KB	*Gene*	GO ID Annotations for molecular function	GO ID Annotations for biological process
Rab-3D	O95716	*RAB3D*	GO:0005515 - protein binding<br>GO:0003924 - GTPase activity	GO:0006887 – exocytosis<br>GO:0009306 - protein secretion<br>GO:0015031 - protein transport
Synaptogyrin-2	O43760	*SYNGR2*	GO:0005515 - protein binding	GO:0045055 - regulated exocytosis
15 kDa phosphoprotein enriched in astrocytes (PEA-15)	Q15121	*PEA15*	GO:0005515 - protein binding	GO:1902042 - negative regulation of extrinsic apoptotic signaling pathway via death domain receptors<br>GO:0000165 - MAPK cascade<br>GO:0046325 - negative regulation of glucose import
ArgBP2	O94875	*SORBS2*	GO:0005515 - protein binding<br>GO:0003723 - RNA binding<br>GO:0005200 - structural constituent of cytoskeleton	GO:0007010 - cytoskeleton organization<br>GO:0007219 - Notch signaling pathway
Beta-COP	P35606	*COPB2*	GO:0005515 - protein binding	GO:0006890 - retrograde vesicle-mediated transport, Golgi to endoplasmic reticulum<br>GO:0006886 - intracellular protein transport
Collagen alpha-2(IV) chain	P08572	*COL4A2*	GO:0030020 - extracellular matrix structural constituent conferring tensile strength	GO:0030198 - extracellular matrix organization<br>GO:0038063 - collagen-activated tyrosine kinase receptor signaling pathway<br>GO:0001525 - angiogenesis
FN	P02751	*FN1*	GO:0005515 - protein binding<br>GO:0005201 - extracellular matrix structural constituent<br>GO:0005178 - integrin binding<br>GO:0005102 - signaling receptor binding	GO:0051702 biological process involved in interaction with symbiont<br>GO:0007155 cell adhesion<br>GO:0034446 substrate adhesion-dependent cell spreading<br>GO:0007160 - cell-matrix adhesion
CTGF	P29279	*CCN2*	GO:0005515 - protein binding<br>GO:0008201 - heparin binding<br>GO:0005178 - integrin binding<br>GO:0005520 - insulin-like growth factor binding	GO:0007155 - cell adhesion<br>GO:0007165 - signal transduction<br>GO:0045597 - positive regulation of cell differentiation<br>GO:0001525 – angiogenesis<br>GO:0007160 - cell-matrix adhesion<br>GO:0008543 - fibroblast growth factor receptor signaling pathway
EF-2	P13639	*EEF2*	GO:0005515 - protein binding<br>GO:0003924 - GTPase activity<br>GO:0003746 - translation elongation factor activity	GO:0006414 - translational elongation
Histone H3.3	P84243	*H3-3A*	GO:0005515 - protein binding<br>GO:0003677 DNA binding<br>GO:0030527 - structural constituent of chromatin	GO:0006334 - nucleosome assembly

Protein name	Uniprot KB	*Gene*	GO ID Annotations for molecular function	GO ID Annotations for biological process
hnRNP D-like	O14979	*HNRNPDL*	GO:0003723 - RNA binding<br>GO:0005515 - protein binding	GO:0010468 - regulation of gene expression
MIF	P14174	*MIF*	GO:0005515 - protein binding<br>GO:0050178 - phenylpyruvate tautomerase activity<br>GO:0005125 - cytokine activity	GO:0070374 - positive regulation of ERK1 and ERK2 cascade<br>GO:0043066 - negative regulation of apoptotic process<br>GO:0043518 - negative regulation of DNA damage response, signal transduction by p53 class mediator<br>GO:0048146 - positive regulation of fibroblast proliferation<br>GO:0001516 - prostaglandin biosynthetic process
NID-1	P14543	*NID1*	GO:0005201 - extracellular matrix structural constituent<br>GO:0005518 - collagen binding<br>GO:0042813 - Wnt receptor activity<br>GO:0043236 - laminin binding	GO:0007160 - cell-matrix adhesion<br>GO:0007155 - cell adhesion<br>GO:0030198 - extracellular matrix organization
RNA-binding protein 34	P42696	*RBM34*	GO:0003723 - RNA binding	GO:0000463 - maturation of LSU-rRNA from tricistronic rRNA transcript (SSU-rRNA, 5.8S rRNA, LSU-rRNA)
Serpin H1	P50454	*SERPINH1*	GO:0005515 - protein binding<br>GO:0004867 - serine-type endopeptidase inhibitor activity<br>GO:0005518 - collagen binding	GO:0006986 - response to unfolded protein<br>GO:0030199 - collagen fibril organization
Transmembrane protein 263	Q8WUH6	*TMEM263*	/	/
XRCC1	P18887	*XRCC1*	GO:0005515 - protein binding<br>GO:0072572 - poly-ADP-D-ribose binding	GO:0006284 - base-excision repair<br>GO:0010836 - negative regulation of protein ADP-ribosylation<br>GO:1903518 - positive regulation of single strand break repair<br>GO:0001666 - response to hypoxia
14-3-3 protein theta	P27348	*YWHAQ*	GO:0005515 - protein binding	GO:0006605 - protein targeting<br>GO:0007165 - signal transduction
ADH1B	P00325	*ADH1B*	GO:0004745 - all-trans-retinol dehydrogenase (NAD+) activity<br>GO:0004022 - alcohol dehydrogenase (NAD+) activity	GO:0001523 - retinoid metabolic process<br>GO:0006066 - alcohol metabolic process<br>GO:0006629 - lipid metabolic process
ADH1C	P00326	*ADH1C*	GO:0004022 - alcohol dehydrogenase (NAD+) activity<br>GO:0004745 - all-trans-retinol dehydrogenase (NAD+) activity	GO:0006066 - alcohol metabolic process<br>GO:0042572 - retinol metabolic process<br>GO:0042573 - retinoic acid metabolic process
ATP synthase subunit O	P48047	*ATP5PO*	GO:0005515 - protein binding<br>GO:0046933 - proton-transporting ATP synthase activity, rotational mechanism	GO:0006754 - ATP biosynthetic process<br>GO:0015986 - proton motive force-driven ATP synthesis
Breast carcinoma-amplified sequence 1	O75363	*BCAS1*	/	GO:0042552 - myelination

Protein name	Uniprot KB	*Gene*	GO ID Annotations for molecular function	GO ID Annotations for biological process
CaCC-1	A8K7I4	*CLCA1*	GO:0005229 - intracellularly calcium-gated chloride channel activity<br>GO:0004222 - metalloendopeptidase activity	GO:1902476 - chloride transmembrane transport<br>GO:0006508 - proteolysis<br>GO:0006816 - calcium ion transport
CA-II	P00918	*CA2*	GO:0004089 - carbonate dehydratase activity<br>GO:0005515 - protein binding	GO:0051453 - regulation of intracellular pH<br>GO:0015670 - carbon dioxide transport<br>GO:2001150 - positive regulation of dipeptide transmembrane transport<br>GO:0002009 - morphogenesis of an epithelium
CE-2	O00748	*CES2*	GO:0106435 - carboxylesterase activity	GO:0006629 - lipid metabolic process<br>GO:0006693 - prostaglandin metabolic process<br>GO:0006805 - xenobiotic metabolic process
CEH	P34913	*EPHX2*	GO:0004301 - epoxide hydrolase activity<br>GO:0016791 - phosphatase activity<br>GO:0033885 - 10-hydroxy-9-(phosphonooxy)octadecanoate phosphatase activity<br>GO:0042577 - lipid phosphatase activity<br>GO:0042803 - protein homodimerization activity	GO:0042632 - cholesterol homeostasis<br>GO:0097176 - epoxide metabolic process<br>GO:0010628 - positive regulation of gene expression<br>GO:0016311 – dephosphorylation<br>GO:0006629 - lipid metabolic process<br>GO:0009636 - response to toxic substance
CgA	P10645	*CHGA*	/	GO:0033604 - negative regulation of catecholamine secretion<br>GO:0042742 - defense response to bacterium<br>GO:0002551 - mast cell chemotaxis
Decorin	P07585	*DCN*	GO:0030021 - extracellular matrix structural constituent conferring compression resistance<br>GO:0005515 - protein binding	GO:0016239 - positive regulation of macroautophagy<br>GO:0010596 - negative regulation of endothelial cell migration<br>GO:0016525 - negative regulation of angiogenesis
Ep-CAM	P16422	*EPCAM*	GO:0005515 - protein binding<br>GO:0044877 - protein-containing complex binding<br>GO:0098641 - cadherin binding involved in cell-cell adhesion	GO:0045944 - positive regulation of transcription by RNA polymerase II<br>GO:0008284 - positive regulation of cell population proliferation<br>GO:0048863 - stem cell differentiation<br>GO:0098609 - cell-cell adhesion
EPLIN	Q9UHB6	*LIMA1*	GO:0005515 - protein binding<br>GO:0051015 - actin filament binding<br>GO:0003779 - actin binding<br>GO:0045296 - cadherin binding	GO:0016477 - cell migration<br>GO:0030299 - intestinal cholesterol absorption<br>GO:0031529 - ruffle organization
Gal-3	P17931	*LGALS3*	GO:0005515 - protein binding<br>GO:0030246 - carbohydrate binding<br>GO:0019863 - IgE binding<br>GO:0043236 - laminin binding<br>GO:0140693 - molecular condensate scaffold activity	GO:0002548 - monocyte chemotaxis<br>GO:0030593 - neutrophil chemotaxis<br>GO:0045806 - negative regulation of endocytosis<br>GO:2001237 - negative regulation of extrinsic apoptotic signaling pathway
IgGFc-binding protein	Q9Y6R7	*FCGBP*	GO:0005515 - protein binding	/
PYY	P10082	*PYY*	GO:0005179 - hormone activity	GO:0007631 - feeding behaviour

Protein name	Uniprot KB	*Gene*	GO ID Annotations for molecular function	GO ID Annotations for biological process
			GO:0001664 - G protein-coupled receptor binding	GO:0007218 - neuropeptide signaling pathway<br>GO:0060575 - intestinal epithelial cell differentiation
Tryptase-1	Q15661	*TPSAB1*	GO:0004252 - serine-type endopeptidase activity<br>GO:0005515 - protein binding	GO:0006508 - proteolysis<br>GO:0022617 - extracellular matrix disassembly<br>GO:0006952 - defense response
UDP-GlcDH	O60701	*UGDH*	GO:0003979 - UDP-glucose 6-dehydrogenase activity	GO:0006065 - UDP-glucuronate biosynthetic process<br>GO:0015012 - heparan sulfate proteoglycan biosynthetic process<br>GO:0030206 - chondroitin sulfate biosynthetic proces
Beta ig-h3	Q15582	*TGFBI*	GO:0005201 - extracellular matrix structural constituent<br>GO:0005515 - protein binding<br>GO:0005178 - integrin binding<br>GO:0050839 - cell adhesion molecule binding	GO:0030198 - extracellular matrix organization<br>GO:0001525 - angiogenesis<br>GO:0002062 - chondrocyte differentiation<br>GO:0007162 - negative regulation of cell adhesion<br>GO:0008283 - cell population proliferation
Galphai2	P04899	*GNAI2*	GO:0005515 - protein binding<br>GO:0003924 - GTPase activity<br>GO:0001664 - G protein-coupled receptor binding	GO:0007165 - signal transduction<br>GO:0001973 - G protein-coupled adenosine receptor signaling pathway<br>GO:0007214 - gamma-aminobutyric acid signaling pathway<br>GO:0008284 - positive regulation of cell population proliferation<br>GO:0051301 - cell division
Adenosine deaminase 2	Q9NZK5	*ADA2*	GO:0004000 - adenosine deaminase activity<br>GO:0008083 - growth factor activity<br>GO:0008201 - heparin binding<br>GO:0031685 - adenosine receptor binding	GO:0006154 - adenosine catabolic process<br>GO:0007165 --signal transduction
Alpha-adducin	P35611	*ADD1*	GO:0003779 - actin binding<br>GO:0005515 - protein binding<br>GO:0003723 - RNA binding<br>GO:0005516 - calmodulin binding	GO:0032092 - positive regulation of protein binding<br>GO:1903142 - positive regulation of establishment of endothelial barrier<br>GO:1903393 - positive regulation of adherent junction organization
Complement C4-B	P0C0L5	*C4B*	GO:0001848 - complement binding<br>GO:0004866 - endopeptidase inhibitor activity<br>GO:0030246 - carbohydrate binding	GO:0006956 - complement activation<br>GO:0006954 - inflammatory response<br>GO:0008228 - opsonization<br>GO:0045087 - innate immune response
HPLPN3	Q96S86	*HAPLN3*	GO:0005540 - hyaluronic acid binding	GO:0001501 - skeletal system development<br>GO:0002052 - positive regulation of neuroblast proliferation<br>GO:0007155 - cell adhesion
MCRIP1	C9JLW8	*MCRIP1*	GO:0005515 - protein binding	GO:0010717 - regulation of epithelial to mesenchymal transition
NDUFV1	P49821	*NDUFV1*	GO:0005515 - protein binding<br>GO:0008137 - NADH dehydrogenase (ubiquinone) activity	GO:0006120 - mitochondrial electron transport, NADH to ubiquinone
VSIG2	Q96IQ7	*VSIG2*	/	GO:0006629 - lipid metabolic process

Protein name	Uniprot KB	*Gene*	GO ID Annotations for molecular function	GO ID Annotations for biological process
ZPR1	O75312	*ZPR1*	GO:0031369 - translation initiation factor binding<br>GO:0008270 - zinc ion binding<br>GO:0005515 - protein binding	GO:0061564 - axon development<br>GO:0010628 - positive regulation of gene expression<br>GO:0042307 - positive regulation of protein import into nucleus<br>GO:0000226 - microtubule cytoskeleton organization<br>GO:0045927 - positive regulation of growth<br>GO:1902742 - apoptotic process involved in development

Table S2. Mean Decrease Accuracy (MDA) scores of the three groups (S, D and H), and average MDA calculated for the 21 proteins selected for the RF classification analysis. The relatively higher MDA scores for each protein are highlighted.

S	D	H	average	
0.039	0.002	0.099	0.046	Galphai2
0.011	0.025	0.091	0.042	CA-II
0.050	0.059	0.006	0.038	GRASP-1
0.034	0.072	0.001	0.036	Sorting nexin-18
0.006	0.033	0.067	0.035	CEH
0.022	0.000	0.083	0.035	ADH1C
0.028	0.043	0.018	0.029	Beta-COP
-0.008	0.017	0.065	0.025	IgGFc-binding protein
-0.008	0.028	0.034	0.018	CaCC-1
0.021	0.003	0.029	0.018	Ep-CAM
0.008	0.028	0.015	0.017	PYY
0.023	0.023	0.004	0.017	Transmembrane protein 263
0.015	0.005	0.022	0.014	ADH1B
0.020	-0.008	0.028	0.014	S100A9
0.009	0.019	0.010	0.012	NID-1
0.026	0.008	0.003	0.012	OGDH-E1
0.012	0.003	0.014	0.010	14-3-3 protein theta
0.002	0.021	0.006	0.010	XRCC1
0.015	-0.003	0.012	0.008	Otase
-0.004	0.009	0.012	0.006	CgA
0.007	0.006	0.002	0.005	MCRIP1

Table S3. Results of the correlation analysis between Dukes stage, budding index and the LFQ abundances of the proteins (n=91) with significant changes between groups. Spearman (Sp) Pearson (Pe) tests were used depending on whether the data were normally distributed or not; *p*-values and r coefficient are reported for each type of tissue (S, D and H). In bold the proteins that contribute to the RF classification.

Protein	S	D
	**Dukes stage** ***p*-value (*r*)**	
Alpha-adducin	0.04 *(0.52) Pe*	
ArgBP2	0.04 *(0.52) Pe*	
Collagen alpha-2(IV) chain	0.03 (*0.53) Sp*	
EC-SOD	0.02 (*0.57) Sp*	
FN	0.03 (*0.55) Pe*	
N-WASP	0.04 *(-0.52) Pe*	
Synaptogyrin-2	0.04 *(-0.52) Pe*	
**14-3-3 protein theta**		**0.04 (*0.51*) *Pe***
Beta ig-h3		0.03 (*-0.55*) *Sp*
**Galphai2**		**0.01 (*0.61*) *Pe***
NASP		0.04 (*-0.52*) *Pe*
VSIG		0.02 (*-0.58*) *Pe*
	**Budding index** ***p*-value (*r*)**	
Gal-3	0.02 *(-0.58) Sp*	
Synaptogyrin-2	0.01 *(-0.60) Sp*	
Alpha-Bcrystallin		0.02 *(-0.58) Pe*
CD2-associated protein		0.02 *(-0.58) Sp*
CTGF		0.005 *(-0.67) Sp*
**OGDH-E1**		0.02 *(0.57) Sp*
PLD3		0.002 *(-0.72) Sp*

Figure S1. Topology of the backbone network. It consisted of 196 nodes with high betweenness centrality values and 1,787 edges. The size of the nodes corresponds to their betweenness centrality values. Pink colored nodes are proteins with levels varied from the statistical analysis.

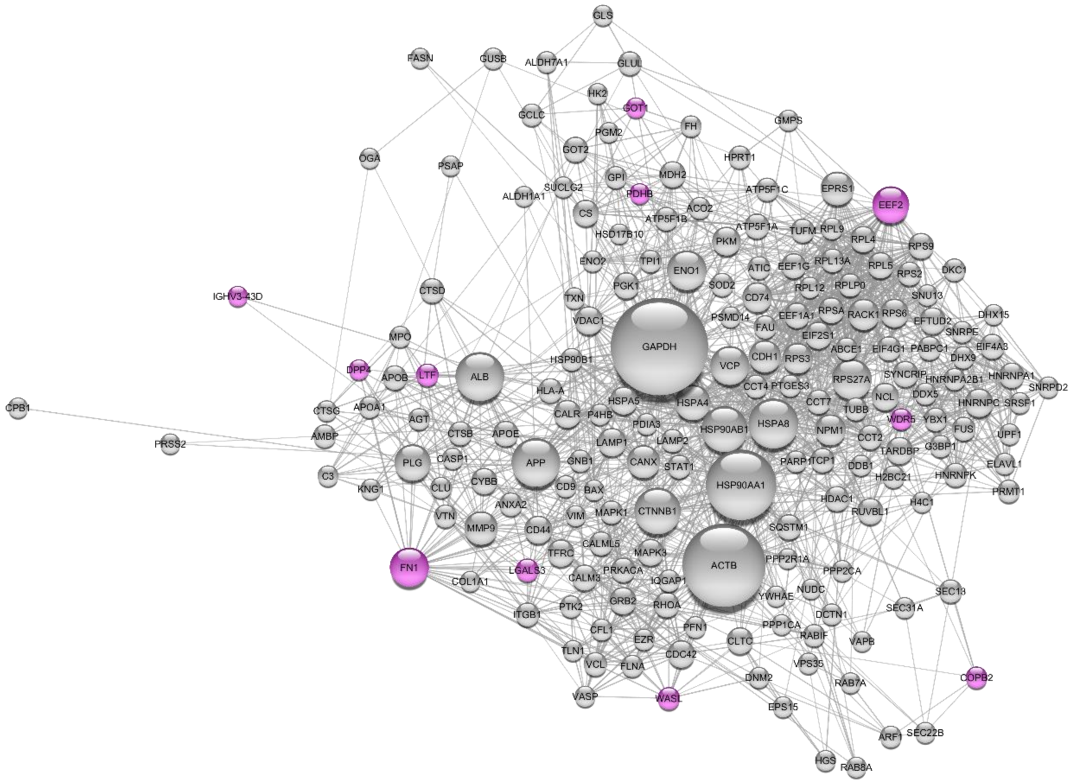

Supplement: Supplementary file 1 [file cells-13-01311-s001.zip › Supplementary_file S2.pdf]
